# Alleviating time poverty among the working poor: a pre-registered longitudinal field experiment

**DOI:** 10.1038/s41598-021-04352-y

**Published:** 2022-01-14

**Authors:** Ashley Whillans, Colin West

**Affiliations:** 1 Negotiations, Organizations & Markets Unit, Harvard Business School, Harvard University, Cambridge, MA USA; 2 Rotman School of Management, University of Toronto, Toronto, Canada

**Keywords:** Human behaviour, Social behaviour

## Abstract

**Abstract:**

Poverty entails more than a scarcity of material resources—it also involves a shortage of time. To examine the causal benefits of reducing time poverty, we conducted a longitudinal field experiment over six consecutive weeks in an urban slum in Kenya with a sample of working mothers, a population who is especially likely to experience severe time poverty. Participants received vouchers for services designed to reduce their burden of unpaid labor. We compared the effect of these vouchers against equivalently valued unconditional cash transfers (UCTs) and a neutral control condition. In contrast to our pre-registered hypotheses, a pre-registered Bayesian ANCOVA indicated that the time-saving, UCT, and control conditions led to similar increases in subjective well-being, reductions in perceived stress, and decreases in relationship conflict (Cohen’s *d*’s ranged from 0.25 to 0.85 during the treatment weeks and from 0.21 to 0.36 at the endline). Exploratory analyses revealed that the time-saving vouchers and UCTs produced these benefits through distinct psychological pathways. We conclude by discussing the implications of these results for economic development initiatives.

**Protocol registration:**

The Stage 1 protocol for this Registered Report was accepted in principle on 27/06/2019. The protocol, as accepted by Nature Human Behaviour, can be found at https://doi.org/10.6084/m9.figshare.c.4368455.

Poverty is associated with lower engagement in preventative health care (even when access is available)^1,2^, lower medication adherence^3^, increased spending on ‘temptation goods’^4^, reduced productivity at work^5^, and lower adoption of useful new technologies (e.g., agricultural innovations)^6^. These seemingly disparate behaviors may share a common feature: they may be driven, in part, by the fact that people living in material poverty also tend be ‘time poor.’ Indeed, poverty is not only a state of material constraints, it also involves temporal constraints. The current study explored whether time poverty reinforces barriers toward economic mobility and contributes to poverty traps.

Consistent with previous research^7–9^, we refer to individuals as ‘time poor’ when they engage in long hours of unpaid work and have no choice but to do so. Time poverty severely affects low-income women living in developing countries. A lack of basic household amenities requires poor women to spend far more time on household production tasks like cooking and cleaning as compared to their richer counterparts^10^. For example, women in Sub-Saharan Africa spend an average of 4.2 h on unpaid work each day^11^. These unpaid household activities can be conceptualized as a kind of tax that individuals, especially women, must pay before undertaking remunerated work. In this project, we propose that reducing time poverty, thereby lowering this personal ‘tax,’ will have direct benefits for subjective well-being, perceived stress, and relationship conflict, as well as indirect benefits for economic well-being.

Despite these potentially far-reaching consequences, there is little understanding of the psychological and economic consequences of the time poverty that often coincides with financial constraints. Traditional economic measurements of poverty often neglect the fact that households living below the poverty line face substantive time deficits (Hirway provides a comprehensive review^12^). Furthermore, aid programs tend to focus on material constraints. Billions of dollars of economic aid have been spent to provide monetary and non-monetary aid to people living in extreme poverty. The most common aid programs include food, livestock, and fertilizer, as well as services such as agricultural training, community health workers, and teachers^13–16^. We suggest that the effectiveness of these aid programs could be increased by considering recipients’ time costs, either by adjusting how aid is delivered or by creating programs directly aimed at reducing recipients’ temporal constraints (Khera provides related arguments^17,18^).

One reason that aid programs may neglect time poverty is the lack of data on time-use amongst the working poor in developing countries. While richer countries have benefited from extensive survey data on time-use, these data are critically absent from countries where time poverty is the most pervasive (Hirway provides a comprehensive review^12^). Despite these limitations, there is some evidence that time poverty may be an important factor in economic development efforts. A large-scale correlational analysis of the Indian Human Development survey, which included 41,554 households in 1503 villages and 971 urban neighborhoods, found that women who owned a cookstove and did not have to fetch wood were healthier and spent more time on income generating activities than women who did not own a cookstove^19^. Of course, this research cannot rule out selection effects: Women with higher wealth or status in their communities might be more likely to own and benefit from appliances such as cookstoves.

One previous study experimentally tested the causal effects of reducing unpaid labor^20^. In this experiment, sixty working adults recruited in Vancouver, Canada were assigned to spend a small windfall of money ($40 CAD) during two consecutive weekends. During one weekend, participants were instructed to spend this windfall in any way that would save them time. During another weekend, participants were instructed to spend this windfall on a material purchase for themselves. After making a time-saving (vs. material) purchase, participants reported greater positive mood, lower negative mood, and lower perceived stress. However, this experiment targeted affluent individuals living in North America, provided a small one-time payment, and assessed immediate mood. It is therefore unclear whether these findings would apply to poverty alleviation efforts.

Given the limited causal evidence in this area, we used a randomized control trial to evaluate the benefits of reducing time poverty. We recruited working women living in Kibera, an urban slum near Nairobi, Kenya. We selected this population because women living in this context face significant material and temporal constraints. In Kibera, working women earn an average of 100–200 KSH ($1–2 USD^21^) per day and spend a median of 42 h on paid labor and 36 h on unpaid labor each week. We randomly assigned women living in this community to receive time-saving vouchers designed to reduce their burden of unpaid labor for three consecutive weeks. Specifically, these vouchers were redeemable for cooking or cleaning services (methodological details below). Based on our pilot data, we expected both time-saving vouchers to provide study participants with an additional 3–7 h each week.

We compared the effect of these time-saving vouchers against equivalently valued unconditional cash transfers (UCTs). We also compared time-saving vouchers and UCTs against a control condition in which participants did not receive aid of any kind.

UCTs have received a great deal of attention as a critical tool for poverty alleviation in developing countries^22,23^. Recent research finds that UCT’s produce significant welfare benefits^22^. For example, in a large-scale field experiment in Kenya (*N* = 1372), households that received UCTs experienced significant improvements in self-reported happiness, life satisfaction, and perceived stress^22^. These well-being benefits persisted for up to 3 years^23^. Cash transfers have also been shown to increase hours of employment, monthly net earnings, and subjective financial well-being when provided to the unemployed^24,25^, and to improve monthly cash earnings when provided to micro-entrepreneurs^26^. Cash transfers also improve empowerment among adolescent girls and young women, as proxied by increased agency and control over decision-making, greater access to financial resources, improved schooling outcomes, decreased teen pregnancy, and better health^27^. Furthermore, the administrative and overhead costs of providing unconditional cash transfers are extremely low. Given the well-documented benefits and low administrative costs, UCTs serve as a stringent standard by which to compare the effectiveness of aid programs designed to save time. Using equivalently valued UCTs as a benchmark, we measured the effectiveness of time-saving services and compared the benefits of reducing time versus financial poverty^28,29^.

Reducing time poverty directly addresses a critical market failure in urban slums. Time poverty is pervasive in this context due to limited infrastructure and a high cost for basic services (e.g., water, sewage, and electricity^30^). People in urban slums also cannot afford to purchase time-saving services. In Kibera, there are several small businesses that offer such services, but they are largely unaffordable. For example, a single load (8 kg) of laundry costs 500 KSH, on average, which equates to over three times the average daily wage. In our pilot data, 76.5% of working women living in Kibera reported “never” paying for laundry services, and 82.4% reported “never” paying for prepared meals from local vendors. Providing cash transfers is unlikely to address this market failure because people do not readily spend money on time-saving services, even when they can afford to do so^20^.

Policymakers are not systematically addressing this market failure, partially because they also undervalue the possible benefits of time-saving services. In an initial pilot study, we asked thirty current and aspiring policymakers from the Harvard Kennedy School of Public Policy how they would allocate 2100 KSH ($21 USD) of aid per recipient to improve the welfare of working women living in Kibera. Only 6% of respondents spontaneously reported that the 2100 KSH should be used to save time for these women. When we explicitly provided respondents with the choice to fund one of three aid programs (an unconditional cash transfer program, an in-kind goods program, or a time-saving program), only four respondents (13%) selected the time-saving program, and twenty-six respondents (87%) chose cash. These findings suggest that both recipients and policymakers undervalue time-saving services.

In contrast, we expected that reducing temporal (vs. financial) poverty would have a positive impact on three critical outcomes: subjective well-being, perceived stress, and relationship conflict. We focused on subjective well-being and perceived stress because these outcomes are linked to economic decision-making^31^. For example, greater positive affect is associated with a range of downstream economic benefits including increased productivity, work performance, and higher earnings^32,33^. Furthermore, stress caused by poverty is linked to short-sighted economic decision-making and excessive risk aversion^34^. We focused on relationship conflict based on existing evidence showing that cash transfers can reduce intimate partner violence^35^. However, there is also some data showing that providing cash windfalls to women may lead to arguments with their partner about how to spend this income, possibly increasing domestic violence^22^. Because gains of time are harder to account for than gains of money^36^ and because time is less fungible than money^37^, we predicted that providing women with time-saving vouchers would be less likely to cause relationship conflict than cash transfers.

As discussed above, recent research finds that receiving cash transfers can have positive benefits for subjective well-being^22,38^, stress^22,39,40^ and intimate partner violence^35,41^. Prior research also finds that time-saving services can have positive benefits for subjective well-being, perceived stress, and relationship conflict^20^. Building on this research, we pre-registered three primary hypotheses to be tested using nine pre-registered comparisons. We predicted that participants who were randomly assigned to receive UCTs or time-saving vouchers would experience positive benefits on each of our three key outcomes at endline compared to participants who were randomly assigned to the control condition. We also predicted that participants assigned to receive time-saving vouchers would experience greater positive benefits on these outcomes compared to participants who received UCTs. To test these hypotheses, we committed to collecting data until we had reached a Bayes Factor > 10 or < 0.010, or until we had reached a total sample size of *N* = 2000 participants across our three conditions.

Specifically, we pre-registered that we would calculate Bayes Factors for each comparison after reaching an initial sample size of *N* = 1200 and, if we found inconclusive results, we would collect an additional 800 participants. Due to the COVID-19 pandemic, we were forced to terminate data collection in March 2020, resulting in a total sample of *N* = 1070. We report Bayes Factors for all nine preregistered comparisons using this sample of *N* = 1070. See the “Methods” section for further detail. Our pre-registered hypotheses are documented below.

- **H**_**1**_: Women who are randomly assigned to receive UCTs for three consecutive weeks will report higher subjective well-being, lower perceived stress, and lower relationship conflict at endline compared to women who are assigned to the control condition and receive no aid of any kind.
- **H**_**2**_: Women who are randomly assigned to receive time-saving services for three consecutive weeks will report higher subjective well-being, lower perceived stress, and lower relationship conflict at endline compared to women who are assigned to the control condition and receive no aid of any kind.
- **H**_**3**_: Women who are randomly assigned to receive time-saving services for three consecutive weeks will report higher subjective well-being, lower perceived stress, and lower relationship conflict at endline compared to women who are assigned to receive equivalently valued UCTs.

## Results

## Pre-registered results

### Pre-processing checks

Before testing our three primary hypotheses, we conducted pre-registered analyses on patterns of attrition and a manipulation check assessing whether the time-saving condition successfully reduced participants’ burden of unpaid labor.

Overall, we observed a low rate of attrition across the 6 weeks of the experiment (*N* = 83; 7.2%). Consistent with our pre-registered plan, we first conducted a chi-square analysis to examine whether there was differential attrition for participants assigned to the control, UCT, or time-saving conditions. In the control condition 34 participants (8.7%) did not complete the study; in the UCT condition 20 participants (5.2%) did not complete the study; and in the time-saving condition 29 participants (7.7%) did not complete the study. These differences were not statistically significant, *X*^2^ (2, *N* = 1153) = 3.86, *p* = 0.145.

As per our pre-registered analysis plan, we then tested for differential attrition along the following baseline characteristics: age, education, marital status, number of people living in the household, number of children living in the household, the household member responsible for financial decision-making, total hours of paid labor in the past 7 days, total hours of unpaid labor in the past 7 days, total income over the past 6 months, total household spending over the past 7 days, depression, subjective well-being, perceived stress, and relationship conflict. Attrition did not differ across any of these characteristics (see Supplementary Tables S1a&b).

The final sample at endline included 1070 participants (*M*_*age*_ = 36.10, *SD* = 9.19*;* 73.0% were married or in a marriage-like relationship; *M*_*children*_ = 3.04, *SD* = 1.43; median baseline weekly income = 1663.35 KSH, *SD* = 1969.54; *M*_*weekly hrs of paid work*_ = 39.83, *SD* = 18.56; *M*_*weekly hrs of unpaid work*_ = 40.79, *SD* = 24.66. See Supplementary Tables S2–S3 for further detail on sample characteristics. Random assignment was successful in balancing conditions on relevant demographic, employment, well-being, and economic characteristics (Supplementary Table S4). Given the success of random assignment and the fact that we did not observe selective attrition, it is unlikely that attrition meaningfully impacted our results.

Next, we conducted a pre-registered manipulation check to determine whether the time-saving condition had a significant impact on participants’ burden of unpaid labor relative to the UCT condition. During each of the treatment weeks, participants reported changes in their burden of unpaid labor in the past 7 days on a scale from − 3 = *decreased a lot* to 0 = *no change* to 3 = *increased a lot*. We created a weighted average of participants’ responses to this item during the treatment weeks (Weeks 3–5). Using this measure, we conducted a Bayesian *t-test* with the null hypothesis that there was no difference between conditions regarding a change in the burden of unpaid labor across treatment weeks (H_0_: δ = 0). The one-sided alternative hypothesis in this analysis states that the time-saving condition led to a reduction in the burden of unpaid labor as compared to the UCT condition (H_A_: δ < 0). Based on Rouder et al. (2009)^42^, we assigned δ a Cauchy prior distribution with *r* = 1/√ 2, truncated to only allow negative effect size values.

Consistent with our a priori predictions, the time-saving condition led participants to experience a significant reduction in their perceived burden of unpaid labor (*M*_*time-saving*_ = − 2.27, *SD* = 0.87, 95% credible interval [− 2.38, − 2.17]) as compared to participants in the UCT condition (*M*_*UCT*_ = − 0.67, *SD* = 1.01, 95% credible interval [− 0.78, − 0.57]). In these analyses, we observed a BF_A, 0_ > 1000, meaning that the data were more than 1000 times more likely under H_A_ than under H_0_. The median resulting posterior distribution for the effect size δ equaled 1.67 (95% credible interval [1.48, 1.86]). This result indicates decisive evidence for H_A_^43^. See Supplementary Table S5 and Fig. S1 for the full results of these analyses.

### Primary analyses

Given these successful pre-processing checks, we conducted our primary pre-registered analyses. Our primary pre-registered analyses examined between condition differences in endline subjective well-being, perceived stress, and relationship conflict controlling for the respective baseline measures. For each of these outcomes, we conducted a Bayesian ANCOVA with the following planned comparisons: UCT versus control condition, time-saving versus control condition, and UCT versus control condition. For each comparison, we calculated a Bayes factor (B_10_) comparing the null model (M_0_, including the baseline measure) against a model (M_1_) that included the condition effect. See “Methods” section for further detail.

In three Bayesian ANCOVAs, we found strong evidence in support of the null hypothesis that there were no differences in endline subjective well-being, perceived stress, or relationship conflict across conditions, controlling for the respective baseline measures. For subjective well-being, the data were 41 times more likely to occur under the null model compared to the model that included the condition effect (*BF*_*10*_ = 0.025). For perceived stress, the data were 70 times more likely under the null model (*BF*_*10*_ = 0.014). For relationship conflict, the data were 36 times more likely under the null model (*BF*_*10*_ = 0.028). Nine pre-registered pairwise comparisons also revealed substantial evidence in favor of the null hypothesis as indicated by Bayes Factors ranging from 0.01 to 0.32 (see Tables 1, 2 for Bayesian ANCOVA results).

**Table 1:** Bayesian model comparisons.

Models	P(M)	P(M|data)	BF_M_	BF_10_	Error %
**Models predicting endline SWB:**					
M_0_: Null model (incl. baseline SWB)	0.500	0.976	40.508	1.000	
M_1_: Condition + baseline SWB	0.500	0.024	0.025	0.025	2.478
**Models predicting endline PSS:**					
M_0_: Null model (incl. baseline PSS)	0.500	0.986	69.725	1.000	
M_1_: Condition + baseline PSS	0.500	0.014	0.014	0.014	1.662
**Models predicting endline conflict:**					
M_0_: Null model (incl. baseline conflict)	0.500	0.973	36.184	1.000	
M_1_: Condition + baseline conflict	0.500	0.027	0.028	0.028	2.111

Reporting the prior model probability, P(M); the posterior model probability, P(M|data); the posterior model odds, BF_M_; and the Bayes Factor indicating the predictive performance of a given model divided by the predictive performance of the null model (BF_10_).

**Table 2:** Bayesian pairwise comparisons.

		Prior odds	Posterior odds	BF_10_	Error %
**Pairwise comparisons on endline SWB**					
Control	UCT	0.587	0.058	0.099	0.002
Control	Time-saving	0.587	0.112	0.191	0.001
UCT	Time-saving	0.587	0.062	0.105	0.002
**Pairwise comparisons on endline PSS**					
Control	UCT	0.587	0.049	0.083	0.002
Control	Time-saving	0.587	0.064	0.109	0.002
UCT	Time-Saving	0.587	0.064	0.110	0.002
**Pairwise comparisons on endline conflict**					
Control	UCT	0.587	0.067	0.114	0.002
Control	Time-saving	0.587	0.189	0.322	< 0.001
UCT	Time-saving	0.587	0.069	0.118	0.002

Individual comparisons are based on a Cauchy prior distribution with an *r*-scale value of 0.3 for comparisons between the UCT condition and the control condition; 0.5 for comparisons between the pre-registered Time-saving condition and the control condition; and 0.4 for comparisons between the UCT and Time-saving conditions.

Our pre-registered analyses did not support our pre-registered hypotheses. These pre-registered analyses found no differences between conditions at endline on the three primary outcomes of interest: subjective well-being, perceived stress, and relationship conflict.

## Exploratory results

We then conducted exploratory analyses to examine longitudinal trends, mechanisms, and individual differences in treatment effects.

### Longitudinal analyses

In contrast to our a priori predictions, all three conditions had significant positive effects on each outcome variable that were similar in magnitude. Collapsing across condition, participants experienced a sizeable baseline-to-endline increase in subjective well-being, from *M* = 2.71, *SD* = 0.68 to *M* = 2.89, *SD* = 0.74, *F*(1,1069) = 46.48, *p* < 0.001, *d* = 0.21; a sizeable reduction in perceived stress, from *M* = 3.24, *SD* = 0.54 to *M* = 3.03, *SD* = 0.61, *F*(1,1069) = 86.32, *p* < 0.001, *d* = 0.29; and a sizeable reduction in relationship conflict, from *M* = 0.94, *SD* = 0.96 to *M* = 0.58, *SD* = 0.79, *F*(1,1069) = 136.87, *p* < 0.001, *d* = 0.36.

**Figure 1:**
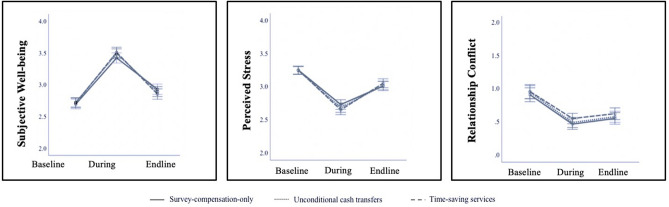
Effect of condition and time point on subjective well-being, perceived stress, and relationship conflict. This figure reports estimate marginal means and 95% confidence intervals by each condition and time point: at baseline (Week 1), during the intervention (weighted average of Weeks 3–5), and at endline (Week 6). As indicated by the confidence intervals that do not overlap, all outcome measures differed significantly from the baseline to endline measurements, suggesting that the positive impact of the condition assignments persisted even after the intervention ended. Outcome measures during the intervention were calculated as a weighted average of participants’ responses in Weeks 3, 4, and 5. For subjective well-being, the measure during the intervention was a weighted average of positive affect and negative affect in Weeks 3–5. The Satisfaction with Life scale was not included because it required a visual aid and was not administered in the phone survey during intervention weeks.

To explore these results further, we conducted a repeated measures ANOVA testing for the effect of condition on each outcome: (1) at baseline, (2) during the intervention, and (3) at endline. Consistent with our pre-registered analyses, we found no significant effect of condition on subjective well-being, perceived stress, or relationship conflict. However, we did find a significant effect of time for each outcome, such that the benefits were greatest during the intervention (Weeks 3–5) but persisted at endline. Post-hoc pairwise comparisons revealed that, relative to baseline, participants in each condition experienced a significant difference on all three outcomes during the intervention and at endline (*ps* < 0.001). We found no significant interaction effects between condition and time. See Fig. 1 and Supplementary Tables S6a–S8c.

### Mechanisms

Consistent with the exploratory analyses specified in our Stage 1 Report, we conducted a series of bootstrapped mediation and moderation analyses to test for three mechanisms by which time-saving vouchers could improve subjective well-being, perceived stress, and relationship conflict during the study. First, time-saving vouchers might produce benefits by reducing the total amount of time spent on unpaid labor. Second, time-saving vouchers might remove especially disliked tasks. Third, time-saving vouchers might enable recipients to spend more time on welfare-enhancing activities such as paid work or socializing.

We found evidence in support of the first mechanism: Time-saving vouchers conferred psychological benefits by reducing unpaid labor. This finding aligns with research showing a robust negative association between unpaid labor and psychological well-being, particularly for working women^44^. The effect of the time-saving condition on perceived stress during the intervention was mediated by a reduction in participants’ burden of unpaid labor (indirect effect = − 0.11 (0.05), 95% CI [− 0.19, − 0.01]). Reducing the burden of unpaid labor did not mediate the effect on subjective well-being (indirect effect = 0.07 (0.06), 95% CI [− 0.04, 0.18]) or relationship conflict (indirect effect = 0.003 (0.05), 95% CI [− 0.09, 0.10]). See Supplementary Tables 9a.

We also conducted mediation and correlation analyses to test for the second and third possible mechanisms. We did not find evidence for either mechanism. Additional time spent on paid work or socializing and the extent to which participants disliked cooking and doing laundry did not predict the benefits of the time-saving vouchers (Supplementary Table S10a–S13b).

Participants in the UCT condition experienced benefits through a distinct mechanism: an increase in ‘cash on hand’ during the treatment weeks (“How much cash [in shillings] does your household have on hand right now?”). Receiving unconditional cash transfers led to an average increase in monthly income of 29% relative to baseline earnings. In turn, increased cash on hand mediated the effect of the UCT condition on subjective wellbeing (indirect effect = − 0.02 (0.01), 95% CI [− 0.05, − 0.001] and perceived stress (indirect effect = 0.02 (0.01), 95% CI [0.001, 0.04]). Cash on hand did not mediate the effect of the UCT condition on relationship conflict (indirect effect = − 0.003 (0.005), 95% CI [− 0.005, 0.02]). These findings suggest that the benefits of UCTs were partially driven by an increase in liquid cash resources and not from an increase in spending. The important role of ‘cash on hand’ for psychological well-being aligns with prior research showing that having readily available cash predicts life satisfaction over and above total income, investments, and debts^45^.

**Figure 2:**
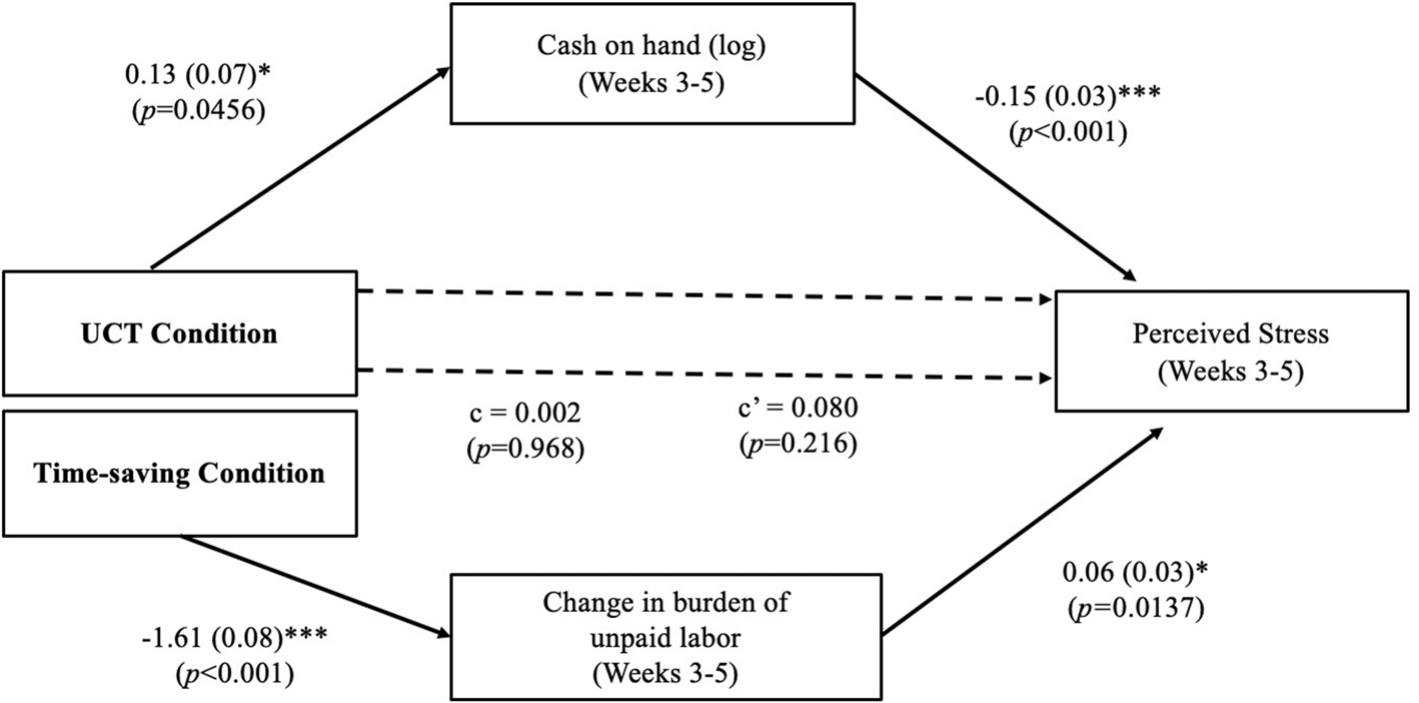
Parallel mediation analysis. Mediating effects of changes in perceived burden of unpaid labor and (log) cash on hand. Reported are standardized coefficients, controlling for baseline perceived stress and baseline income. Cash on hand (log), change in burden of unpaid labor, and total spending (log) during the treatment weeks were analyzed as parallel mediators. The indirect effect of cash on hand (log) was significant, indirect effect = 0.02 (0.01), 95% CI [0.004, 0.04]. The indirect effect of the change in burden of unpaid labor was significant, indirect effect = − 0.10 (0.04), 95% CI [− 0.19, − 0.02]. The indirect effect of total spending (log) was not significant, indirect effect = 0.001 (0.004), 95% CI [− 0.005, 0.010]. The total effects of conditions (time-saving = 1, UCT = 0) are reported as *c*; direct effects are reported as *c’*. See Table S9a for further detail. *p < 0.05, **p < 0.01, ***p < 0.001.

We examined these distinct mechanisms for the benefits of the time-saving versus UCT conditions using parallel mediation models. In each model, the burden of unpaid labor, cash on hand (log), and total spending (log) during the treatment weeks were analyzed simultaneously as parallel mediators, while controlling for baseline cash on hand (log), baseline income (log), and the respective baseline outcome measure. See Fig. 2 and Supplementary Tables S9a.

### Individual characteristics

Consistent with the exploratory analyses specified in our Stage 1 Report, we also conducted a series of regression analyses to assess whether the benefits of each condition differed based on participant characteristics at baseline. It is possible that the benefits of time-saving vouchers might be strongest for participants who are experiencing the greatest stress at baseline or for participants with work skills that allow them to take on additional paid labor. There might also be greater benefits of time-saving vouchers for participants who are employed in occupations that are flexible and have a greater availability of work, such as occupations that involve short-term contracts or micro-enterprises.

We found no evidence that the following baseline characteristics significantly influenced the benefits of the time-saving condition relative to UCTs: education level, occupation, household size, income, well-being, perceived stress, relationship conflict, or risk of depression. We did observe a moderating effect of micro-enterprise ownership. Participants who were micro-entrepreneurs (i.e., who reported owning a business or a portion of a business; 30% of the sample) experienced greater benefits from cash transfers compared to time-saving vouchers. Specifically, micro-entrepreneurs who were randomly assigned to receive cash transfers experienced a larger decrease in perceived stress at endline controlling for baseline compared to micro-entrepreneurs who received time-saving services. See Supplementary Tables S14a–S15b.

## Discussion

We conducted a pre-registered, highly powered field experiment over 6 weeks with a sample of 1070 working mothers living in Kibera, an urban informal settlement in Nairobi, Kenya. The results from our pre-registered Bayesian analyses did not support our a priori predictions. Time-saving services did not increase subjective well-being, lower perceived stress, or reduce relationship conflict more than UCTs, and time-saving services and UCT’s did not outperform the control condition. Instead, and in contrast to our a priori hypotheses, our pre-registered Bayesian analyses provide evidence that receiving three consecutive weeks of time-saving services, cash transfers, or being assigned to the control condition had significant and statistically similar benefits for subjective well-being, perceived stress, and relationship conflict.

Although these benefits were greatest during the intervention period, exploratory analyses suggest that these benefits persisted after the intervention ended. Follow-up analyses show that all conditions continued to have a positive impact on subjective well-being, stress, and relationship conflict ranging from *d* = 0.21 to *d* = 0.36 at endline. However, the effects were attenuated at endline, which is consistent with previous research on cash transfers suggesting that their impacts weaken across time^22^. See Supplemental Tables S6c, S7c, and S8c. Why did the time-saving, UCT, and control conditions have similar benefits for subjective well-being?

Relative to baseline earnings, participants in the UCT condition experienced a 33% average increase in their total income during the intervention period, whereas participants in the control condition experienced a 14% increase in their total income from the payments that they received for completing the study-related surveys. It is therefore possible that the benefits we observed across the UCT, control, and time-saving conditions could be a consequence of the financial deprivation that our participants faced at baseline. Prior to the intervention, participants earned a median income of 1250 KSH per week and had a median total savings of 0 KSH. Our data suggest that under such circumstances, even small cash infusions, the provision of limited additional paid work, and time-saving services may have a positive psychological impact.

As the control condition did not differ from the time-saving or UCT conditions, it is also possible that participants in the control condition derived some psychological benefit from the money that they earned from completing the phone surveys and an additional psychological benefit due to *working* for this money; thus, yielding similar psychological benefits to the UCT and time-saving conditions despite receiving less money (and less valuable compensation) in total. This possibility aligns with the results of a field experiment in Kenya showing that working for pay improved well-being relative to waiting for an equivalently valued windfall^46^. Further research is needed to understand the psychological effects of receiving cash transfers of various sizes and the benefits of providing additional work relative to cash and non-cash aid. It is also possible that the repeated interactions with field officers could have produced some of the benefits observed in the study. Future research should explore the potential benefits of social connection interventions for promoting well-being in this context.

It is also possible that the duration of the field experiment was too short to observe differential effects across conditions. To provide an initial test of whether time-saving services promote the well-being of women living in Kibera, participants received UCTs, time-saving services or they were randomly assigned to the control condition for three consecutive weeks. During this period, we found evidence that women did benefit from time-saving services to a similar extent as UCTs. The cumulative benefits over a longer period may be substantially different if participants are better able to make plans for upcoming cash and time infusions. For example, longer-term time-saving services may provide increasing marginal returns and more persistent benefits if people spend their additional time on repeated rewarding activities like learning a new skill, applying for new jobs, establishing a new business, or volunteering^47^.

Building on these propositions, future research should test the relative efficacy of larger time-saving and cash transfer interventions over longer durations, especially given the fact that many existing studies of UCT’s start with large initial transfers and/or monthly transfers across years^22,48,49^. For example, future research could replicate and extend our results by comparing time-saving technologies—such as rainwater collection technology that can save up to 8 h of unpaid labor per day—against the provision of sustained cash transfers^50^.

As noted above, we did observe persistent effects of each intervention on our pre-registered outcomes of interest; however, these effects were attenuated at Week 6 (see Fig. 1). This attenuation is consistent with prior research on cash transfers^22^ and is suggestive of more limited long-term impacts of these interventions. These data speak to the importance of better understanding the *persistent benefits* of cash transfers and other in-kind aid like time-saving services to further understand the possible policy relevant implications of this work.

Another critical factor that could help to explain why we observed no differences between conditions at endline is that we were unable to test the effect of UCTs or time-saving services against a neutral control condition. Given our focus on subjective well-being, it was necessary to bring participants into the lab to fill out surveys. It was therefore necessary to pay participants for completing the study, to adhere to ethical guidelines and reduce the possibility of differential attrition across conditions. Consequently, the statistical similarity between the benefits of the UCT and control conditions reveals an open area for future research: the amount of cash or work necessary to create sustainable changes in subjective well-being. Relatedly, additional research should explore whether the benefits of time-saving services are greater when these services do not incur any time cost. In our study, participants had to travel short distances to pick up their meals and to pick-up and drop-off laundry. Future research should examine whether reducing these small frictions would bolster the benefits of time-saving services.

In our pilot studies, working women in Kibera reported that meal and laundry services were highly desirable: these services would save a significant amount of time and would reduce a disliked chore. Although working women in this sample spent a considerable amount of time cooking and doing laundry each week, and reported that these tasks were unenjoyable, very few women spent money to reduce the burden of these chores: In our pilot data, 100% reported “never” spending money on laundry services and 82.4% “never” purchased take-out. These findings are consistent with research conducted in North America showing that working adults often fail to make time-saving purchases. In one representative study of working Americans, only 15% reported spending money to buy time^20^. In another study, only 2% of working Canadian parents reported that they would spend a windfall of $100 on a purchase that would save time^51^. Together, these data suggest that people often fail to spend money on time-saving services, even when they are experiencing time stress. See^36^ for a review of related research.

Of course, it is likely that spending money on time-saving services is not the most pressing concern for women living in Kibera and therefore the low base-rates of spending on these services in this context does not reflect a suboptimal valuation of time, but rather a sensible spending decision due to significant financial constraints. As a result, it is important to understand the beliefs that policy makers hold about the value of time-saving services, given that they could provide aid or make other bureaucratic changes that could save constituents time. Given the small scope of our pilot data, additional research should systematically explore the extent to which policy makers undervalue the time of people who are living in poverty.

Future research could explore the extent to which working women in this context *themselves* undervalue time and broaden the range of time-saving services studied to include other services that our pilot data suggests may be helpful, such as providing delivery services for local business owners or the direct delivery of clean drinking water. Future research could also explore the benefits of time-saving services adapted to the unique sources of time stress faced among populations whose temporal constraints are caused by different factors.

We selected the interventions used in this study based on extensive piloting to ensure that the time-saving interventions were desirable and helpful for the study population. We also worked with a local non-profit organization, the Human Needs Project, to implement these interventions through their previously established community center, Kibera Town Center. This non-profit had been operating in the local community for seven years prior to the implementation of this study. Given the success of Kibera Town Center to offer these services to the low-income population living in Kibera before our research group began studying the causal impacts of time-saving services, we believe that that these time-saving interventions would be scalable in other urban informal settlements where employed adults work long hours and are financially constrained. If community centers like Kibera Town Center become more common within urban informal settlements, this could lower the cost of the already subsidized services provided by these non-profits, thereby assisting in scaling these interventions more broadly.

Although our study provides substantial evidence that time-saving services do not improve well-being beyond UCTs or a control condition that included survey compensation, our exploratory analyses provide insights that shape our understanding of when the benefits of time-saving services (and UCT’s) are likely to emerge. Exploratory pathway analyses suggest that UCTs and time-saving services confer welfare benefits through distinct mechanisms.

UCT’s reduced perceived stress by increasing the availability of ‘cash on hand.’ Consistent with past research^45^, immediately available cash appeared to provide a sense of financial safety that was critical for the emotional well-being of the women in our study. In contrast, time-saving services reduced perceived stress by lowering participants’ perceived burden of unpaid labor. This finding is consistent with prior research showing that the benefits of time-saving purchases are driven by the perception of reduced daily demands^20,51^.

We also found initial exploratory evidence that female microentrepreneurs with school-aged children living at home derived greater psychological benefits from cash (vs. time-saving vouchers), experiencing lower stress at endline after receiving 1500 KSH. However, microentrepreneurs in the time-saving condition earned the most money. Microentrepreneurs in the time-saving condition earned 343 KSH more than microentrepreneurs in the UCT condition and 309 KSH more than those in the control condition. These comparisons were statistically significant. Building on these results, future work should examine the relative effects of cash transfers (vs. time-saving services) on the well-being and earnings of microentrepreneurs.

The insights generated from this field experiment could have implications for promoting welfare and economic mobility among chronically stressed populations. Participants in this study were financially and temporally impoverished. They were also at a high risk for clinical depression. Twenty-percent of participants reported depression scores on a validated measure (CES-D) that were indicative of being at risk for clinical depression^52^. These rates are consistent with research on the prevalence of depression among people living in poverty (rates ranging from 15–45%)^53^. This chronic stress and risk of depression may perpetuate poverty traps—overlapping economic, environmental, and psychological conditions that make it difficult to escape poverty unless multiple constraints are relieved simultaneously^54–57^. Therefore, reducing stress and depression such as through the provision of repeated payments of cash, time-saving vouchers, or additional work may all be important pathways towards alleviating poverty.

Rigorous testing is needed to develop effective methods of reducing stress and depression for individuals living in severe poverty. For instance, a recent study found that psychotherapy alone had no measurable effect on the psychological well-being of chronically poor individuals living in rural Kenya, whereas cash transfers led to significant improvements in mental health^58^. Furthering this work, our results show that freeing up temporal resources, providing additional cash, or providing additional work may be similarly beneficial strategies for reducing stress among working mothers living in material poverty. Future research could apply similar experimental methods to compare the efficacy of all these strategies for the reduction of stress, with possible downstream consequences for depressive symptomologies and physical health.

One potential limitation of the individual randomization approach taken in this study is that participants could have known each other. To minimize concerns over potential spill-over effects, we took several precautions.

First, we ran the study through an established research center. The Busara Centre for Behavioral Economics runs multiple studies in Kibera at a given time, many of which involve the collection of survey data, as well as cash and in-kind compensation. Therefore, even if a participant knew others in the study, there would be no way to distinguish these participants from participants in any of Busara’s other ongoing studies. Thus, implementing our study through the Busara Centre for Behavioral Economics ensured that it would be very unlikely for participants to directly compare themselves against other people in a different condition.

Second, we took precautions to ensure that participants did not interact with each other. During the intervention period, only participants who received time-saving vouchers visited Kibera Town Center. In contrast, participants who were assigned to the UCT and pure control conditions only visited Kibera Town Center to complete the baseline and endline surveys. During the baseline and endline surveys, we randomized participants at the session level. For example, on Monday mornings, only participants in the control condition completed baseline or endline surveys; participants in the time-saving and UCT conditions were assigned to other sessions. As a result, any interactions were limited to participants assigned to the same condition.

Finally, we measured spillovers through a funnel-debriefing procedure that occurred after participants had completed the final endline surveys. In this debriefing, participants answered three questions to identify possible spillovers. First, participants answered whether they knew anyone participating in the study. Second, participants answered the name of this person to validate that this person was in fact another participant in our study. Third, we asked an open-ended question about what the other participant received as compensation. As per our Pre-Registered Stage 1 Report, we defined “spillover participants” as participants who correctly identified that they knew another participant who received a different form of compensation.

Attesting to the success of these three measures to minimize potential spill-overs, only *N* = 32 [2.8%] of participants correctly identified knowing another person who received a different form of compensation (*N* = 9 were correctly identified in the control condition, *N* = 10 were correctly identified in the UCT condition, and *N* = 13 were correctly identified in the time-saving condition; these rates did not significantly differ by condition, *X*^2^ (2,1,153) = 0.97, *p* = 0.615. We also ran all our pre-registered analyses controlling for an indicator variable of spillovers and the results were unchanged. We can therefore conclude that spillovers were unlikely to have altered the interpretation of our results.

## Conclusion

Policymakers and researchers in economic development tend to focus on alleviating tangible and financial constraints^13,14^, neglecting resources of time or, in many instances, exacerbating time poverty by requiring recipients of aid programs to travel long distances, wait in lines, and fill out arduous paperwork (see Whillans, Giurge, & West, 2020 for a review^50^). Despite being neglected, our data show that reducing time poverty confers similar psychological benefits as UCTs and a control condition that involved paid surveys. These results highlight the need to conduct additional research on the efficacy of time-saving programs and investments.

Future work could involve the provision of time-saving services, time-saving technologies, and infrastructure improvements that save time. The laundry and prepared meal services offered in this study represent two viable time-saving services among many, including improved transportation services to reduce commute times as well as improved cookstoves and water collection technologies. Other technologies may have time-saving elements such as solar-powered appliances that provide off-the-grid households with additional daylight hours to be more productive or engage in leisure activities. More extensive time-tracking, especially in developing countries, is an important step towards evaluating the full range of economic and psychological benefits produced by these infrastructure and technological investments^12^.

In this research, we focused on working mothers in an urban slum in Nairobi because this population is especially likely to face severe financial and temporal constraints. However, time poverty is a pervasive and growing concern around the world, affecting a large and diverse swath of humanity. Data from the Gallup World Poll suggests that stress is rising for people around the world—and it is driven, in part, by the increased demands on our time^59^. A better understanding of time poverty is needed to find more effective and sustainable ways to improve economic and psychological well-being for people living all over the world.

## Methods

This research was approved by the ethics committee at the Harvard Business School (HBS-IRB18-0905) and the Kenya Medical Research Institute (Protocol No. Non-Kemri 629). All participants provided informed consent. Additionally, this research was performed in accordance with all relevant guidelines and regulations for human subjects’ research.

We recruited participants through the Busara Center for Behavioral Economics, a research organization based in Nairobi, Kenya. Busara has a dedicated participant pool of over 15,000 people living in nearby informal settlements, enabling efficient recruitment of working mothers living below the poverty line. The study was implemented from the Kibera Town Center (KTC), a facility located in Kibera and operated by the Human Needs Project. Kibera is the largest informal settlement nearby Nairobi, Kenya, with an estimated 200,000 inhabitants. Based on similar research conducted with Busara^22^, we expected low attrition of around 10%. Consistent with these estimates, we observed low rates of attrition (7.2%).

Women who lived no further than a 30-min walk from Kibera Town Centre were recruited via text message to participate in a 5-min eligibility phone call. This requirement ensured that accessing KTC did not impose a significant time cost. To participate, respondents had to be 18 years of age or older (the legal age of consent in Kenya), provide informed consent, and work for pay at least twenty-five hours per week. To reduce attrition, we only recruited working mothers with at least one child enrolled in school and living at home. These inclusion criteria increased the likelihood that participants would remain in their current residence and complete the study in its entirety. Most women in Busara’s subject pool sent their children to school, therefore we did not expect this eligibility criteria to be a limiting factor. Consistent with this expectation, we excluded only 3.2% of interested respondents based on this criterion.

Based on pilot research, we chose two time-saving vouchers for use in our experiment: prepared meal and laundry services (see below for more information). To ensure that these time-saving vouchers reduced participants’ existing burdens of unpaid labor, we excluded women who reported that they “always” used laundry and/or prepared meal services. Similarly, we excluded respondents who spent fewer than 3 h per week cooking and fewer than 3 h per week completing laundry.

To facilitate data collection, respondents had to have a working cell phone that was not shared with another household member. Over 90% of Kibera residents have their own phone^22^, thus we did not expect to exclude many respondents on this criterion. Consistent with this expectation, we did not exclude any participants based on this criterion. To ensure that the time-saving services meaningfully reduced the burden of unpaid labor, we excluded participants with seven or more individuals living in their household. Lastly, we excluded all participants who did not complete our primary endline measures. Prior to the start of data collection, we added an exclusion criterion that was not originally included in our Stage 1 Report: participants were excluded if they reported food allergies to the meals provided in the time-saving condition. We excluded 2.4% of interested respondents based on this criterion. See Supplementary Table S16 for more detail on the number of exclusions made based on each of these criteria.

As we used validated scale measures that restricted the range of participants’ responses, we did not define or identify outliers. We conducted our proposed pre-registered analyses using all the data that we collected from eligible participants. Based on recently published research conducted through Busara^22^, we expected our variables to be normally distributed.

### Study timeline

**Figure 3:**
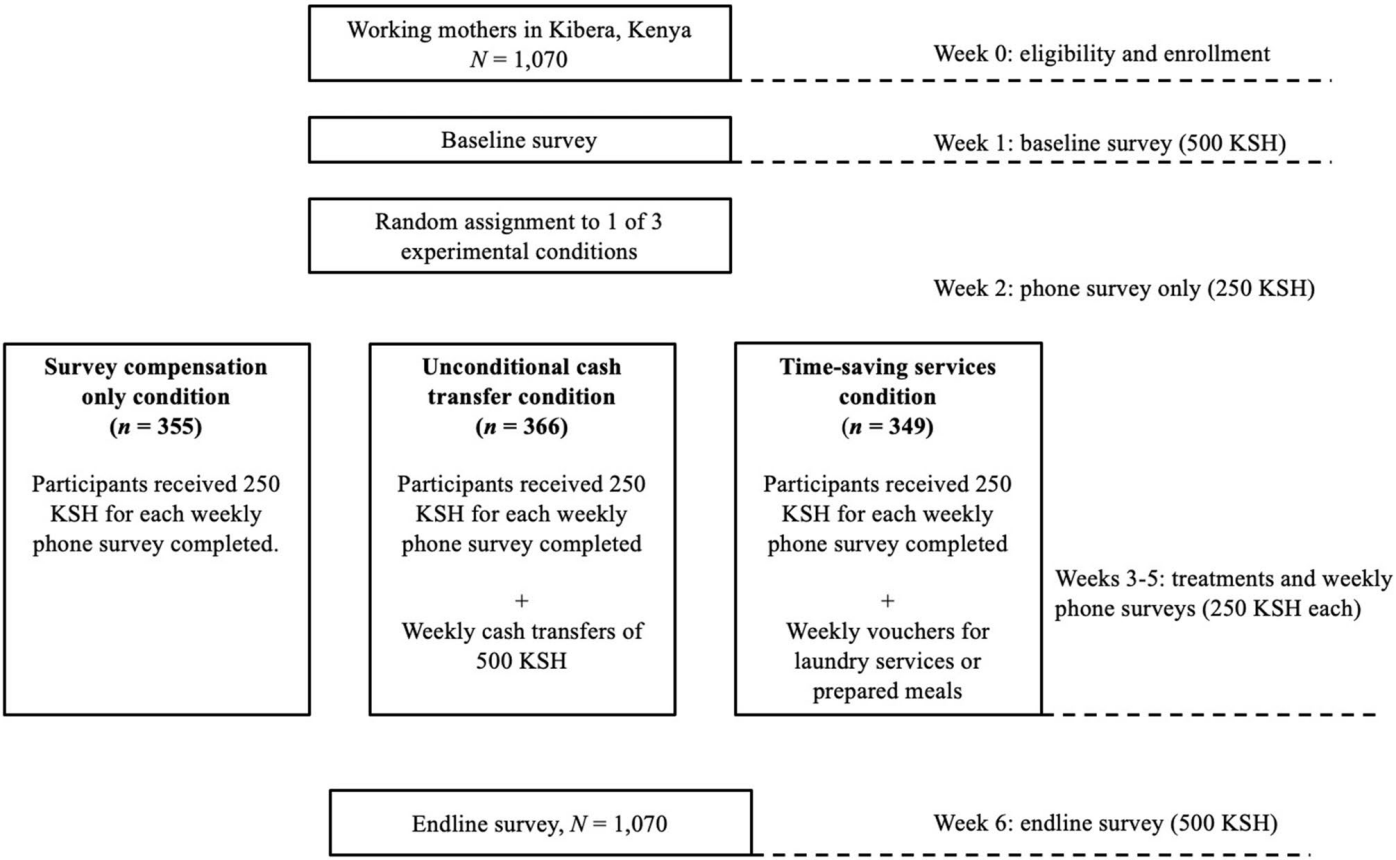
Overview of study design and timeline.

This study included a baseline survey, weekly phone surveys, and an endline survey containing identical pre-registered measures to the baseline. See Fig. 3 for study flow. Data was collected in nine overlapping waves, with each wave lasting 6 weeks. The first survey wave began in September 2019 and the final survey wave ended in March 2020.

As described in our Stage 1 Report, we had planned to collect weekly text message data. However, very few participants opened the weekly text message surveys. Thus, we terminated text message data collection after the first three waves of data collection. As described in our Stage 1 Report, we had also planned to conduct three follow-up phone surveys to track the persistence of any observed treatment effects. Due to high attrition, we terminated these phone surveys after Wave 1. Critically, these protocol changes did not affect any of our pre-registered analyses^60^.

Following from related research^22^, we collected granular data on participants’ affective experiences, stress, time-use, and household consumption throughout the experiment. All participants received compensation for completion of the baseline and endline surveys (500 KSH each) and weekly phone surveys (250 KSH each). Participants in the first three waves of data collection received compensation for completing the text message surveys (25 KSH each).

The baseline survey was conducted in a lab setting (Week 1). Eligible participants were invited to the Kibera Town Centre to provide consent and complete the baseline survey, including the primary pre-registered measures: subjective well-being, perceived stress, and relationship conflict. Participants then completed a variety of exploratory and demographic measures (See the Open Science Framework page for full surveys).

While related studies on UCT’s have collected baseline and endline data by traveling to participants’ homes^22^, we collected data in a lab setting to maximize the number of participants that we could recruit and to minimize the costs associated with data collection by reducing commutes to participants’ homes. Indeed, research on UCTs has noted the high costs of collecting follow-up data in participants’ homes, especially in longitudinal studies where participants frequently move, which was the case in our study population of interest^61,62^.

After completing the baseline survey, participants were randomly assigned between-subjects to one of two treatment conditions or a control condition (1 = time-saving, 2 = UCT, 3 = control). Using the “sample” function in R, we generated a random integer between the values of 1 and 3 by running the following code for each participant: treat < -sample (1:3, 1).

Starting in Week 3, participants who were randomly assigned to one of the two treatment conditions received (1) time-saving services or (2) equivalently-valued unconditional cash transfers. Participants received one of these windfalls every week for three consecutive weeks (Week 3–5). The time-saving and UCT conditions were matched in terms of their cost-to-administer, thereby holding constant the total amount of aid that was disbursed (500 KSH/week).

In Week 6, all participants were invited back to KTC to complete the endline survey, which included identical measures of subjective well-being, stress, and relationship conflict. To ensure that we could collect endline data from participants who were unwilling or unable to come to the Kibera Town Center (e.g., due to forced lockdowns of the COVID-19 pandemic), we modified the pre-registered protocol to provide participants with the option of completing a modified endline survey over the phone. See Supplementary Table S17 for the demographic characteristics of participants who completed the endline survey in-person (vs. over the phone).

For all data collections, trained field officers guided participants through our measures in Swahili. Participants could also complete the measures in English. As was originally noted in the Supplemental Materials of our Stage 1 Report, participants chose their preferred language. This decision ensured that every participant—including participants with limited reading, writing, and numeracy skills—were able to comprehend and correctly complete instructions and measures. Field officers were blind to condition for baseline and endline data collection. Phone surveys were not performed blind to condition since we included a manipulation check asking about how the time-saving vouchers or UCTs affected participants’ burden of unpaid labor in each week. None of the field officers were told about the hypotheses of the study.

#### Details on time-saving vouchers

To develop the time-saving vouchers, we selected services likely to have the greatest benefits for our target population. We conducted a pilot study to identify local services that met the following criteria for working women in Kibera: the services (1) saved a significant amount of time, (2) replaced chores that are unpleasant, and (3) replaced chores that did not involve significant social interaction (i.e., women typically engaged in these chores alone). Based on these criteria, we selected prepared meals and laundry services (see Supplemental Information, Supplemental Results section of our Stage 1 Report for the results of our pilot). For all three treatment weeks, participants who were assigned to the time-saving condition received either prepared meals (two meal varieties alternated across weeks) or laundry services.

#### Condition 1: time-saving vouchers (*N* = 349)

The cost to provide each of these time-saving services was 500 KSH per week. Based on our pilot data, 500 KSH worth of these services eliminated a significant amount of unpaid labor among our target population (3–7 h per week on average; See Supplemental Information, Supplemental Results section of our Stage 1 Report). Building on prior research, we sought to amplify the possible benefits of the time-saving vouchers by reminding participants about the specific amount of time that they would save^63^ and by asking them to make detailed plans for this additional time^64,65^.

#### Condition 2: unconditional cash transfers (*N* = 366)

The weekly cash transfer was 500 KSH. Therefore, participants received 1500 KSH in cash transfers during the treatment period, which amounted to a median income increase of 33% relative to baseline earnings.

#### Condition 3: control (*N* = 355)

Participants received no windfalls of any kind. This condition provided a benchmark for evaluating the effectiveness of the treatment conditions on our key outcomes of interest.

#### Exploratory condition: time-saving vouchers, no planning (*N* = 365)

We included an exploratory condition that was not originally described in our Stage 1 protocol. This condition was identical to Condition 1 with two key changes: (1) We did not remind participants that the laundry and prepared meal services would save time and (2) We did not ask participants how they would spend this additional time. This condition allowed us to explicitly test whether time-saving services were more effective if participants were asked to think about the fact that they were saving time and to plan for having additional free time. This condition was collected alongside the pre-registered conditions for our own conceptual learning and interest. We collected these data without knowing that our data collection would be terminated early due to the COVID-19 pandemic. This condition is purely exploratory; we did not include this condition in any of the analyses reported in the main manuscript and this condition is not discussed further in the main text. For details and analyses of this additional exploratory condition, please see Supplementary Information.

### Manipulation check (T2)

To ensure that the time-saving services reduced burdens of unpaid labor compared to the UCT condition, we asked all participants assigned to our experimental conditions to complete the following question each treatment week: “Over the PAST 7 DAYS, to what extent did receiving [prepared meals/laundry/cash transfers] affect your burden of unpaid labor?” Participants indicated their response on a scale from − 3 = *Decreased my burden of unpaid labor a lot*, 0 = *Did not change my burden of unpaid labor*; 3 = *Increased my burden of unpaid labor a lot*. We combined and averaged participants’ responses across the three treatment weeks.

### Primary measures (T1 and T2)

To measure subjective well-being at baseline and endline, participants completed (a) the 12-item Schedule of Positive Affect and Negative Affect (SPANE^66^: PA_T1_ α = 0.91; NA_T1_ α = 0.91; PA_T2_ α = 0.92; NA_T2_ α = 0.92), and (b) the 5-item Satisfaction with Life Scale (SWLS^67^). Based on past research, we defined subjective well-being (SWB) as a combination of high positive affect (PA), low negative affect (NA), and high satisfaction with life (SWL)^68–70^. Each of these measures were highly correlated at baseline (*rs*_B_ > 0.51, *ps* ≤ 0.01) and endline (*r*_*E*_ > 0.50, *ps* < 0.01). We therefore created a composite measure at both time points by combining PA (averaged), SWL (averaged) and NA (averaged and reverse-coded). We deviated from the pre-registered protocol to allow participants in the final four survey waves to complete the endline survey over the phone. This change was implemented in response to the COVID-19 pandemic.

The over-the-phone survey was identical to the in-person survey, with one exception. In the endline phone survey, we were unable to administer the Satisfaction with Life (SWL) scale because it required a visual aid. For participants who chose to complete the endline measures over the phone, we calculated a SWB composite measure using participants’ responses to the Positive Affect (PA) and Negative Affect (NA) items only. We report our pre-registered treatment effect analyses both on the full sample of participants looking only at the SWB composite comprised of PA and NA (*N* = 1070) and the sub-sample of participants who completed the endline in-person and completed all three sub-components of SWB (*N* = 764). Results are substantially unchanged. See Supplementary Tables S18–S19.

To measure perceived stress, participants completed the 10-item Perceived Stress Scale (PSS^71,72^) at baseline and endline. The PSS conceptualizes perceived stress as a lack of control over important life outcomes. Previous research suggests that both time-saving services and UCTs increase perceived control over daily events^73^. Our focus on this definition of stress addresses recent calls from researchers to focus on specific elements of stress, since stress as an overall concept has become too broad to be useful^74^. We created a composite measure of perceived stress at both time points by taking the average of all items of the PSS (PSS_B_ α = 0.77; PSS_E_ α = 0.82).

To measure relationship conflict at baseline and endline, participants completed the 9-item negative interaction scale of the network of relationship inventory^75^. We created a composite of relationship conflict at both time points by taking the average of all nine items (RelationshipConflict_B_ α = 0.95; RelationshipConflict_E_ α = 0.95). As noted in the Supplemental Materials of our Stage 1 Report, if participants reported that they were not married or in a marriage-like relationship they completed the identical measures of relationship conflict by answering with respect to their closest social relationship (e.g., sister, mother). To preserve statistical power, we report treatment effects on the combined measure of relationship conflict (collapsing across reports of conflict with romantic partners and close social relationships). We also examine the effects for the sub-sample who were married or in a marriage-like relationship (*N* = 604). Results are substantively unchanged. See Supplementary Tables S20–S21.

### Pre-registered analysis pipeline

#### Pre-processing checks

Before testing our primary hypotheses, we conducted chi-square analyses to ensure that we did not have differential attrition across conditions. This pre-processing check was successful. There were no systematic differences in attrition by condition (see “Results” section). For each of the pre-registered outcomes, we examined and reported differences at baseline between participants who dropped out of the study and those who completed the full study. There were no systematic differences. We also conducted a Bayesian ANCOVA to examine whether participants in the time-saving condition felt less burdened by unpaid labor during the intervention period as compared to participants in the UCT condition. Our data reached BF > 10 for this comparison, indicating a successful manipulation check (see “Results” section).

### Analytic plan for pre-registered, confirmatory hypotheses

#### Overview

We tested each of our pre-registered hypotheses using Bayesian ANCOVA analyses. We first tested for differences between the UCT and control conditions at endline. We then tested for differences between the time-saving and control conditions at endline. Lastly, we tested for differences between the UCT and time-saving conditions at endline. We conducted separate Bayesian ANCOVA analyses to test for differences in subjective well-being, perceived stress, and relationship conflict at endline, controlling for the respective baseline measure in each analysis. We planned to run up to *N* = 1200 participants across our three conditions. If we did not reach a Bayes Factor < 0.10 or > 10.00 on our key outcomes of interest for each comparison, we planned to collect an additional 800 participants for a total sample of *N* = 2000 participants across our three conditions (i.e., UCTs, time-saving, and control). We did not reach this final data collection stopping point due to the COVID-19 pandemic. See below.

#### UCT versus control

Following the approach advocated for by Rouder and colleagues^42^, we calculated the Bayes factor B_10_ by comparing M_1_, the model with the condition effect, and M_0_, the null model. The null model M_0_ had the prior placed at point 0. To specify our Bayesian priors, we placed a half Cauchy prior with an *r*-scale value of 0.3 on the condition effect in M_1_. This prior was selected based on research suggesting a small to medium effect size of cash transfers on well-being, stress, and relationship conflict^21^. Based on a simulation under the assumption of *d* = 0.30 for the difference between the UCT and control conditions, there was a 95% probability that B_10_ would exceed 10.00 under this procedure with a per condition sample size of *N* = 666. The analysis was performed using JASP. B_10_ did not exceed 10.00 across our three dependent measures, therefore we could not conclude that the data presented strong evidence for differences in subjective well-being, stress, and relationship conflict between the UCT and control conditions. B_10_ did not drop below 0.10, therefore we could not conclude that the data presented strong evidence against differences between the UCT and control conditions by journal standards. B_10_ was equivalent to or below 0.11 across all three analyses (Table 2). Well-established Bayesian reference guides indicate that these results provide “substantial” evidence in support of the null hypothesis^43,76^. Thus, we concluded that the data presented substantial evidence against differences between the UCT and control conditions.

#### Time-saving voucher versus control

To specify our Bayesian priors, we placed a half Cauchy prior with an *r-*scale value of 0.5 on the condition effect in M_1_. This prior was selected based on research suggesting a medium effect of time-saving services on subjective well-being, perceived stress, and relationship conflict^20^. Based on a simulation under the assumption of *d* = 0.50 for the difference between the time-saving and control conditions, there was a 99% probability B_10_ would exceed 10.00 under this procedure with a per condition sample size of *N* = 666. B_10_ did not exceed 10.00 across our three dependent measures, therefore we could not conclude that the data presented strong evidence for differences in well-being, stress, and relationship conflict between the time-saving and control conditions. B_10_ did not drop below 0.10, therefore we could not conclude that the data presented strong evidence against differences between conditions by journal standards. B_10_ was equivalent to or below 0.32 across all three analyses (Table 2). Well-established Bayesian reference guides indicate that these results provide “substantial” evidence in support of the null hypothesis^43,76^. Thus, we concluded that the data presented substantial evidence against differences between conditions.

#### UCT versus time-saving voucher

To specify our Bayesian priors, we placed a half Cauchy prior with an *r*-scale value of 0.4 on the condition effect in M_1_. This prior was selected based on research suggesting a medium effect of receiving time-saving services compared to receiving material goods on subjective well-being and perceived stress^20^. Based on a simulation under the assumption of *d* = 0.40 for the difference between the time-saving and UCT conditions, there was a 99% probability B_10_ would exceed 10 under this procedure with a per condition sample size of *N* = 666. B_10_ did not exceed 10.00 for each of our three dependent measures, therefore we could not conclude that the data presented strong evidence for differences in well-being, stress, and relationship conflict between the UCT and time-saving conditions. B_10_ did not drop below 0.10, therefore we could not conclude that the data presented strong evidence against differences between the UCT and time-saving conditions by journal standards. B_10_ was equivalent to or below 0.12 across all three analyses (Table 2). Well-established Bayesian reference guides indicate that these results provide “substantial” evidence in support of the null hypothesis^43,76^. Thus, we concluded that the data presented substantial evidence against condition differences.

Pre-registration guidelines from this journal required us to confirm that we would collect data until we reached a Bayes Factor < 0.10 or > 10.00 on our key outcomes (subjective well-being, perceived stress, and relationship conflict) for each of our three comparisons of interest (UCT vs. control, time-saving vs. control, and UCT vs. time-saving). In our Stage 1 report, we proposed that we would collect data from an initial twelve hundred participants (*N* = 400 per condition). After collecting data from twelve-hundred participants, we planned to conduct each of the nine Bayesian ANCOVA analyses; three between-condition comparisons for each of the three primary outcomes of interest. If we found inconclusive results, we planned to collect an additional 800 participants sampled evenly across our three conditions for a total of *N* = 2000 participants (or *N* = 666) participants per condition). When all 2,000 participants had completed the study, we committed to re-running all of the pre-specified analyses described above.

After running two-thousand participants, in the unlikely event that our Bayesian results remained inconclusive, we planned to stop data collection and report the results. At this large sample size, inconclusive results would indicate that our treatment conditions were unlikely to provide a welfare improvement, or that our time-saving vouchers would be unlikely to provide a large welfare improvement over UCTs, and that studies with even larger samples would be necessary to provide more definitive results.

Due to the COVID-19 pandemic, we were forced to terminate data collection in March 2020 with a sample of *N* = 1070 participants. Administering the time-saving intervention and measuring our key outcomes of interest required substantial in-person interaction between study staff and participants. In accordance with public health guidelines in Kenya, we terminated data collection before our first pre-registered stopping point. We were unable to collect additional participants due to the ongoing public health threat posed by the COVID-19 pandemic. Thus, all analyses reported in the main manuscript were conducted on this sample of *N* = 1070.

In total, 1550 participants completed baseline measures before data collection was terminated. Of these participants, we collected end line data from 1435 participants, including 1070 participants across our three pre-registered conditions and 365 participants in the additional exploratory time-saving condition conducted for our own interest described above (see Supplementary Information for further details on this condition).

Despite not achieving our pre-registered sample size, we believe the data collected provides robust evidence in support of the null hypothesis across each of our pre-registered analyses. In a follow-up post-hoc power analysis, with the final sample of *N* = 1070, we obtained 98% power to detect our pre-specified effect size of *d* = 0.30 for the comparison between the UCTs vs. control conditions, we obtained 99% power to detect our pre-specified effect size of *d* = 0.50 for the comparison between time-saving vs. control, and we obtained 99% power to detect our pre-specified effect of *d* = 0.40 for the comparison between the UCTs vs. time-saving conditions. Thus, additional data collection was not only unethical due to continued safety concerns but would be highly unlikely to substantively alter our pre-registered results.

### Exploratory analyses

#### Exploratory outcome measures

To further examine the welfare effects of UCTs and time-saving vouchers, we collected additional indicators of economic well-being. At baseline and endline, after completing the pre-registered measures, participants responded to a self-reported measure of subjective financial well-being^77^ as well as a series of items measuring objective financial well-being (adapted from the Federal Reserve Board Survey of Household Economics and Decision Making)^78^. Results were inconclusive and are not discussed further.

#### Longitudinal measures

Throughout the experiment, we collected detailed measures of time-use, consumption, subjective well-being, perceived stress, and relationship conflict (see Open Science Framework for exact questions used). These measures were collected weekly via phone surveys before and during the intervention period (Week 2 through 5). As described above, we did not collect text message data or conduct follow-up surveys due to attrition.

Patterns of time-use were measured once per week during phone surveys. Through a structured interview process, we collected data for the past 7 days on the amount of time spent on various activities, including unpaid labor. We also collected consumption data, including a detailed list of all recalled expenditures over the past 7 days. Lastly, we collected abbreviated measures of well-being, stress, and relationship conflict. Due to attrition, we did not collect an additional measure of well-being via a series of text messages during the experiment.

#### Negative externalities

It is possible that time-saving vouchers could have negative externalities. The time that women save by receiving meals or laundry could be seen by other household members as a fungible resource, thereby increasing the amount of time that women in our sample spend completing unpaid labor for friends and extended family (versus for their own household). As a result, we could not rule out the possibility that participants who received time-saving vouchers might interact less with their friends and family, undermining subjective well-being. Time-saving services could also increase jealousy among extended family and friends and decrease the quality of respondents’ social interactions during the study. To examine these possibilities, our baseline and endline surveys included exploratory questions about time spent with friends and family and satisfaction with these relationships over the course of the study. Our analyses did not provide evidence that time-saving services negatively impacted time spent with friends and family or relationship quality over the course of the study (see “Results” section).

We also explored possible negative externalities of receiving the unconditional cash transfers, including increases in temptation spending (e.g., consumption of alcohol and tobacco), gambling behavior, and a reduction in the motivation to work^79^. We did not observe evidence of these possibilities and these exploratory results are not discussed further.

#### Mechanisms

In our Stage 1 Report, we proposed three possible mechanisms for the subjective well-being benefits of time-saving vouchers. Time-saving vouchers could increase well-being over the course of the study by (1) reducing the total number of hours spent engaging in unpaid labor, (2) removing disliked tasks, or (3) enabling people to spend more of their time engaged in welfare producing activities (e.g., paid work or socializing^32,80^). In the current study, we provided preliminary evidence for each of these possibilities (see “Results” section).

#### Individual differences

The benefits of time-saving vouchers might be strongest for respondents with work skills that allow them to take on additional paid labor. We tested for this possibility by examining whether treatment effects varied by level of education and sector of employment. The benefits of time-saving vouchers might also be strongest in contexts where there is a lot of market work available or where this work primarily consists of short-term contracts or micro-enterprises. We tested for this possibility by examining whether treatment effects varied depending on the nature (i.e., flexibility) of participants’ primary source of income. We found evidence for a moderating effect of micro-entrepreneurship (see “Results” section).

Because the analyses reported in this section were highly exploratory in nature and involved complex moderation and mediation analyses, deviating from the Stage 1 Report, we did not report the strength of the evidence for each of these analyses utilizing a Bayesian approach. See Table 3 for the descriptive statistics of all outcomes at baseline, during the study, and at endline. No data other than the pilot data included at Stage 1 was collected prior to the date of being issued an *acceptance in principle*. The AIP was issued on June 27, 2019. As indicated in the Study Data File, data collection began September 23, 2019.

**Table 3:** Descriptive statistics by condition for subjective well-being (SWB), perceived stress (PSS), and relationship conflict at baseline (week 1), during the interventions (Weeks 3–5, weighted average), and at endline (Week 6).

Time	Condition (n)	SWB *M* (*SD*)	PSS *M* (*SD*)	Conflict *M* (*SD*)
Baseline	Control	2.71 (0.67)	3.25 (0.54)	0.92 (0.96)
	UCT	2.72 (0.72)	3.24 (0.55)	0.93 (0.99)
	Time-saving	2.71 (0.66)	3.24 (0.55)	0.96 (0.95)
During	Control	3.43 (0.73)	2.72 (0.67)	0.47 (0.63)
	UCT	3.50 (0.71)	2.67 (0.63)	0.48 (0.70)
	Time-saving	3.51 (0.74)	2.65 (0.66)	0.54 (0.79)
Endline	Control	2.92 (0.74)	3.02 (0.62)	0.54 (0.79)
	UCT	2.89 (0.75)	3.02 (0.61)	0.58 (0.81)
	Time-saving	2.85 (0.73)	3.05 (0.61)	0.63 (0.77)

Reporting means and standard deviations for each condition at each time point: baseline (Week 1), during the intervention (weighted average of Weeks 3–5), and endline (Week 6). Outcome measures during the intervention were calculated as a weighted average of participants’ responses in Weeks 3, 4, and 5. For subjective well-being, the measure during the intervention was a weighted average of positive affect and negative affect in Weeks 3–5. The Satisfaction with Life Scale was not included in this measure because it required a visual aid and therefore was not administered during the intervention weeks via the phone survey. To be included, participants had to complete 1 or more phone surveys. Endline includes in-person endlines and endlines conducted via phone.

### Supplementary Information

Supplementary Information.

## Data Availability

The datasets generated and analyzed for this project are available through the Open Science Framework: https://osf.io/kpbzd/?view_only=29ecca19774748c28d3e2b0b3741adc8.

## Code availability

Custom code that supports the findings of this study are also available through the Open Science Framework: https://osf.io/kpbzd/?view_only=29ecca19774748c28d3e2b0b3741adc8.
