# Supplementary material for: Alleviating time poverty among the working poor: a pre-registered longitudinal field experiment
Source: Sci Rep. 2022 Jan 14;12:719. doi: 10.1038/s41598-021-04352-y (PMC8760253; doi:10.1038/s41598-021-04352-y)
Supplement: Supplementary file 1 — Supplementary Information. [file 41598_2021_4352_MOESM1_ESM.pdf]

Supplementary information for

Alleviating Time Poverty Among the Working Poor: A Pre-Registered Longitudinal Field Experiment

Ashley Whillans (awhillans@hbs.edu)
Colin West

This file includes:

Section 1: Sample size deviation from Stage 1 registered report (page SM2)
Section 2: Baseline sample characteristics (pages SM3 – SM7)
Section 3: Supplementary methods and results for manipulation check (page SM8)
Section 4: Exploratory results (pages SM9 – SM21)

Lab logs, raw data, and code are available on Open Science Network:
https://osf.io/kpbzd/?view_only=29ecca19774748c28d3e2b0b3741adc8

Section 1: Sample size deviation from Stage 1 registered report

We made one notable change from our pre-registered plan: our data collection stopping point. We specified an endline sample size of $N = 1{,}200$ for the pre-registered Bayesian analyses testing differences in subjective well-being, perceived stress, and relationship conflict across the three pre-registered conditions (Time-Saving Vouchers vs. Unconditional Cash Transfers vs. Control Condition). Additionally, we planned to collect a further 800 participants if we did not observe Bayes Factors < 0.10 or > 10.00 on each of our primary comparisons.

However, after reaching an endline sample size of $N = 1{,}070$ in March 2020 across our three pre-registered conditions of interest, we were forced to terminate data collection due to the COVID-19 pandemic (we collected N=1,435 in total when including data from 365 participants assigned to the additional exploratory time-saving voucher condition described below). We conducted our pre-registered Bayesian ANCOVAs with this sample of N=1,070.

The three omnibus tests of condition on subjective well-being (SWB), perceived stress (PSS), and relationship conflict (conflict) all reached our pre-registered threshold of BF < 0.10. However, we did not reach this threshold on 7 of 9 pre-registered pairwise comparisons between individual conditions. In these comparisons, we observed the following Bayes Factors: BF=0.09 (control vs UCT, effects on SWB); BF=0.19 (control vs time-saving, effects on SWB); BF=0.11 (UCT vs time-saving, effects on SWB); BF=0.08 (control vs UCT, effects on PSS); BF=0.11 (control vs time-saving, effects on PSS); BF=0.11 (UCT vs time-saving, effects on PSS); BF=0.11 (control vs UCT, effects on conflict); BF=0.32 (control vs time-saving, effects on conflict); BF=0.12 (UCT vs time-saving, effects on conflict). Well-established Bayesian reference guides indicate that these results provide “substantial” to “strong” evidence in support of the null hypothesis (Kass & Raferty, 1995). Yet, most of our pairwise comparisons did not reach the pre-registered threshold of BF < 0.10 or >10.00 (see Table 2 of the main manuscript).

We pre-registered that we would collect an additional 800 participants if we did not reach the threshold for Bayes Factors of BF < 0.10 or >10.00. However, due to the COVID-19 pandemic, it was not possible or ethical to continue data collection. Therefore, we terminated data collection in March 2020 in accordance with public health guidelines in Kenya.

Section 2: Baseline sample characteristics

Table S1a. Baseline differences between Attritors (did not completed the full study, (n = 83) and Remainers (n = 1,070).

Variable (measured at baseline)	Attritors Mean (SD)	Remainers Mean (SD)	Difference statistics
Age	34.41 (7.70)	36.10 (9.19)	$t(1148)=1.63$, $p=0.103$
Education (% completed primary school)	77.1%	78.6%	$X^2(N=1, 1153)=0.10$, $p=0.750$
% married or marriage-like relationship	74.7%	73.0%	$X^2(N=1, 1151)=0.11$, $p=0.735$
Household size (total # of people)	4.48 (1.60)	4.68 (1.55)	$t(1151)=1.12$, $p=0.265$
Number of children in the household	3.01 (1.34)	3.04 (1.43)	$t(1151)=0.18$, $p=0.858$
% responsible for household financial decisions	50.6%	50.1%	$X^2(N=1, 1153)=0.01$, $p=0.929$
Hours of paid labor in past 7 days	41.47 (18.12)	39.83 (18.56)	$t(1151)=0.78$, $p=0.437$
Hours of unpaid labor in past 7 days	35.42 (21.89)	40.79 (24.66)	$t(1151)=1.92$, $p=0.055$
Personal income in past 6 months	KSH 38,394 (30,003)	KSH 39,557 (47,243)	$t(1151)=0.22$, $p=0.825$
Household spending in the past 7 days	KSH 3,794 (5,834)	KSH 3,397 (3,767)	$t(1151)=0.88$, $p=0.377$
Baseline depression (CES-D; 1- 4 scale)	2.21 (0.50)	2.24 (0.48)	$t(1151)=0.49$, $p=0.624$
Baseline SWB (1 – 5 scale)	2.77 (0.74)	2.71 (0.68)	$t(1151)=0.85$, $p=0.397$
Baseline PSS (1 – 5 scale)	3.24 (0.55)	3.24 (0.54)	$t(1151)=-0.08$, $p=0.933$
Baseline relationship conflict[a] (0 – 4 scale)	0.95 (1.01)	0.94 (0.96)	$t(1151)=0.10$, $p=0.923$

Notes. Reporting means and standard deviations for respondent characteristics at baseline. Time spent on paid and unpaid labor is measured as a percentage of total time reported for the past 7 days. To adjust for multiple comparisons, we used Bonferroni correction. Using this correction, the significance level for these comparisons is $p \leq 0.004$. Therefore, we can determine that Attritors did not significantly differ from Remainers on any of these baseline characteristics.

Table S1b. Pre-Registered Condition X Baseline Characteristics to Predict Attritors (*n* = 83)

Variable (measured at baseline)	Condition x Variable (Model 1)	Condition x Variable (Model 2)	Overall Model Statistics
Age	*B*=-0.002 (0.03), *p*=0.941	*B*=-0.02 (0.04), *p*=0.619	X^2 (5, 1,550) = 7.58, *p*=0.181
Education (% completed primary school)	*B*=0.37 (0.62), *p*=0.548	*B*=-0.38 (0.80), *p*=0.632	X^2 (5, 1,153) = 5.22, *p*=0.390
% married or marriage-like relationship	*B*=0.97 (0.68), *p*=0.156	*B*=0.99 (0.73), *p*=0.179	X^2 (5, 1,153) = 6.71, *p*=0.243
Household size (total # of people)	*B*= -0.07 (0.17), *p*=0.708	*B*=0.15 (0.21), *p*=0.490	X^2 (5, 1,153) = 6.46, *p*=0.264
Number of children in the household	*B*=-0.30 (0.19), *p*=0.129	*B*=-0.09 (0.23), *p*=0.714	X^2 (5, 1,153) = 6.71, *p*=0.243
% responsible for household financial decisions	*B*=0.47 (0.54), *p*=0.378	*B*=0.85 (0.62), *p*=0.174	X^2 (5, 1,153) = 6.02, *p*=0.304
Hours of paid labor in past 7 days	*B*=0.007 (0.02), *p*=0.654	*B*=0.006 (0.02), *p*=0.740	X^2 (5, 1,153) = 4.91, *p*=0.427
Hours of unpaid labor in past 7 days	*B*=0.02 (0.01), *p*=0.149	*B*=0.02 (0.01), *p*=0.277	X^2 (5, 1,153) = 10.38, *p*=0.065
Personal income in past 6 months	*B*=0.001 (0.01), *p*=0.812	*B*=0.001 (0.001), *p*=0.566	X^2 (5, 1,153) = 4.46, *p*=0.485
Household spending in the past 7 days	*B*=0.001 (0.001), *p*=0.108	*B*=0.001 (0.001), *p*=0.140	X^2 (5, 1,153) = 10.60, *p*=0.060
Baseline depression (CES-D; 1- 4 scale)	*B*=-1.07 (0.057), *p*=0.058	*B*=-0.40 (0.63), *p*=0.524	X^2 (5, 1,153) = 8.03, *p*=0.155
Baseline SWB (1 – 5 scale)	*B*=0.14 (0.39), *p*=0.532	*B*=0.34 (0.43), *p*=0.433	X^2 (5, 1,153) = 5.39, *p*=0.370
Baseline PSS (1 – 5 scale)	*B*=-0.96 (0.49), *p*=0.119	*B*=-0.23 (0.56), *p*=0.682	X^2 (5, 1,153) = 8.30, *p*=0.141
Baseline relationship conflict[a] (0 – 4 scale)	*B*=-0.33 (0.27), *p*=0.222	*B*=0.52 (0.38), *p*=0.176	X^2 (5, 1,153) = 10.60, *p*=0.060

Notes. Reporting means and standard deviations for respondent characteristics at baseline. Time spent on paid and unpaid labor is measured as a percentage of total time reported for the past 7 days. Given the dichotomous nature of the outcome measure, binary logistic regression was used for this analysis. The first interaction term represents Control/UCT vs. Time-saving and the second interaction represents Control/Time-Saving vs. UCT conditions. As indicated by the overall model statistics, none of the overall regression models were significant; thus, the results of interaction models should be interpreted with caution.

Table S2. Sample characteristics at baseline

Variable	Sample characteristics<br>Mean (SD), Range
Age	*M*=35.98 (9.10), *RG*: 20-69
Education (% completed primary school)	78.5%
% married or marriage-like relationship	73.1%
Household size (total # of people)	*M*=4.67 (1.56), *RG*: 1-12
Number of children in the household	*M*=3.04 (1.43), *RG*:1-11
% responsible for financial decisions	50.1%
Hours of paid labor in past 7 days	*M*=40.07 (18.07), *RG*: 0-60+
Hours of unpaid labor in past 7 days	*M*=40.40 (24.50), *RG*: 1-150
Personal income in past 6 months	*M*=39,473 KSH (46,208), *RG*:-30K-888K
Household spending in the past 7 days	*M*=3,425 (3,950), *RG*: 0-52,100
Baseline depression (CES-D; 1- 4 scale)	*M*=2.24 (0.48), *RG*: 1.05-3.75
Baseline SWB (0 – 5 scale)	*M*=2.71 (0.68), *RG*: 1.13-4.87
Baseline PSS (1 – 5 scale)	*M*=3.24 (0.54), *RG*: 1.90-4.70
Baseline relationship conflict (0 – 4 scale)	*M*=0.94 (0.96), *RG*: 0-4.00

Note. 1,000 Kenyan Shillings (KSH) = 9.90 USD (conversion rate as of January 1, 2020). Thus, women in this sample reported making approx. $428.20 USD in the past six months. For women who reported that they did not have a romantic partner, they responded to the items measuring relationship conflict with respect to their closest personal relationship.

Table S3. Participant occupations

Occupation category	Total number (% of baseline sample)
All sales jobs	470 (41.7%)
Trades	50 (4.4%)
Personal services	472 (41.8%)
Casual laborer	81 (7.2%)
Childcare, education, and healthcare services	55 (4.9%)

Note. At baseline, participants provided an open-ended response to the question: "What is your primary job?" This question focused on their occupation. How this person was paid (i.e., salary-based, task-based, or hour/daily) and whether they owned a share in their business (micro-enterprise ownership) were coded separately. Responses were coded into 5 categories: all sales jobs (includes selling produce, meals, clothing, and other consumer goods; working at a kiosk); trades (includes cooks, tailors, construction workers, carpenters, electricians, artisans, and all other skilled labor); personal services (includes hairdressers, restaurant staff, drivers, house cleaners, and washing clothes); casual laborer (includes temporary workers, wage laborer and kibarua); childcare, education, and healthcare services (includes daycare workers, teachers, school administrators, and community health workers). If participants mentioned more than one job, their job code was determined based on the first job they described. Job code was marked as missing if a participant's response could not be understood ($N = 25$).

Table S4. Baseline characteristics, by pre-registered condition assignment

	Control (*N*=389)	UCT (*N*=386)	Time-Saving (*N*=378)	Model Statistics
Age	36.98 (9.64)	35.47 (8.85)	35.47 (8.70)	$F(2, 1147)=3.55, p=0.029$
Education (% completed primary school)	76.9%	79.5%	79.1%	$X^2(N=2, 1153)=0.94, p=0.624$
% married or marriage-like relationship	74.3%	69.7%	75.4%	$X^2(N=2, 1153)=3.58, p=0.167$
Household size (total # of people)	4.71 (1.60)	4.67 (1.57)	4.61 (1.50)	$F(2, 1150)=0.41, p=0.665$
Number of children in the household	3.17 (1.53)	2.97 (1.39)	2.97 (1.34)	$F(2, 1150)=2.47, p=0.085$
% responsible for financial decisions	50.4%	53.9%	46.0%	$X^2(N=2, 1153)=4.73, p=0.094$
Hrs of paid labor in past 7 days	39.97 (18.17)	40.47 (18.27)	39.75 (17.82)	$F(2, 1149)=0.16, p=0.852$
Hrs of unpaid labor in past 7 days	40.54 (25.46)	39.81 (23.90)	40.86 (24.14)	$F(2, 1150)=0.19, p=0.830$
Personal income in past 6 months	KSH 40,510 (47,682)	KSH 36,671 (29,589)	KSH 41,267 (57,256)	$F(2, 1150)=1.09, p=0.336$
Household spending in the past 7 days	KSH 3,380 (3,800)	KSH 3,513 (4,099)	KSH 3,381 (3,953)	$F(2, 1150)=0.15, p=0.865$
Baseline depression (CES-D; 1- 4 scale)	2.24 (0.48)	2.25 (0.50)	2.23 (0.47)	$F(2, 1150)=0.14, p=0.866$
Baseline SWB (0 – 5 scale)	2.71 (0.67)	2.72 (0.72)	2.71 (0.66)	$F(2, 1150)=0.04, p=0.963$
Baseline PSS (1 – 5 scale)	3.25 (0.54)	3.23 (0.54)	3.24 (0.55)	$F(2, 1150)=0.09, p=0.914$
Baseline relationship conflict[a] (0 – 4 scale)	0.92 (0.96)	0.93 (0.99)	0.96 (0.96)	$F(2, 1150)=0.17, p=0.842$

Note. Reporting means, standard deviations, and statistics testing for differences by condition. To adjust for multiple comparisons, we used a Bonferroni correction. With this correction, the significance level for each comparison is $p \leq 0.004$. Thus, we find no significant differences by condition at baseline, which supports our assertion that random assignment was successful.

Section 3: Supplementary methods and results for manipulation check

As a manipulation check, we tested for differences between the UCT and time-saving conditions with respect to 'change in perceived burden of unpaid labor' during the intervention period. In three consecutive weekly phone surveys during the treatment (Weeks 3-5), participants in the UCT and time-saving conditions were asked: "Over the past 7 days, to what extent did receiving [cash / prepared meals / laundry services] affect your burden of unpaid labor (-3=decreased my burden of unpaid labor a lot, 0=did not change my burden of unpaid labor, 3=increased my burden of unpaid labor a lot)? Participants in the control condition were not asked this question since they received no windfalls.

A critical assumption of this research is that participants in the time-saving condition reported experiencing a lower burden of unpaid labor as compared to participants in the UCT condition. Therefore, we conducted a Bayesian independent samples *t-test* (one-sided). This assumption was confirmed. We find a Bayes factor of $BF_{10} > 1000$ (error % < 0.001), which is very strong evidence in support the hypothesis that time-saving services reduce participants burden of unpaid labor (Tables S2-S3, Figure S1).

Table S5. Change in perceived burden of unpaid labor, by condition.

					**95% Credible Interval**	
**Group**	**N**	**Mean**	**SD**	**SE**	**Lower**	**Upper**
UCT	353	-0.67	1.01	0.05	-0.78	-0.57
Time-Saving	288	-2.27	0.87	0.05	-2.37	-2.17

Figure S1. Prior/posterior distribution density plot: Bayesian independent samples *t-test* results for change in perceived burden of unpaid labor.

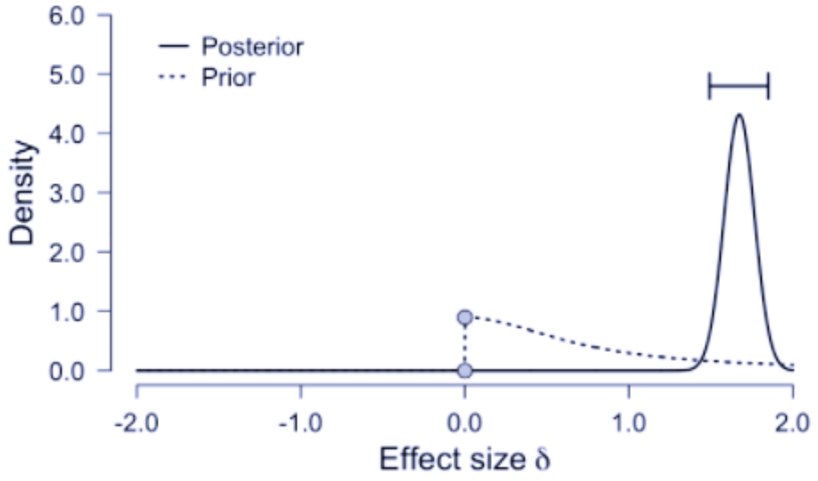

Note. Prior and posterior distributions for effect size, δ. Prior uses a one-sided Cauchy distribution with $r = 1/\sqrt{2}$.

Section 4: Exploratory analyses

We conducted exploratory analyses to examine the effects of condition assignment over the course of the experiment, including effects during the intervention (Weeks 3-5). We explored the mechanisms underlying the observed differences in in subjective well-being, perceived stress, and relationship conflict at three time points: baseline, during the intervention, and endline. Lastly, we examined individual differences in treatment effects based on baseline characteristics including level of education, occupation, microenterprise ownership, household size, income, subjective well-being, perceived stress, relationship conflict, and risk of depression.

For all exploratory analyses, we conduct two sets of analyses. First, we conduct these analyses with three pre-registered conditions (*N*=1,070). Second, we conduct these analyses with data from an additional exploratory time-saving condition described below (*N*=365). All exploratory results in the main text are reported using data from the three pre-registered conditions only.

> **Pre-registered time-saving vouchers condition (*N*=349)**. Participants received either prepared meals or laundry services once per week for three consecutive weeks. To possibly amplify the benefits, participants were asked to make a plan for how they would spend the additional time they had as a result of receiving these time-saving vouchers. Prior to teach treatment week, participants provided an open-ended response to the following question: "Next week, you will receive a [prepared meal service / laundry service] designed to save you time. How do you plan to spend this additional free time?" We then asked participants follow-up questions to increase the specificity of their plans: "Where will you complete this activity / these activities?"; "Who will you complete activity / these activities with?"

> **Additional exploratory time-saving vouchers condition (*N*=365).** This condition was identical to the condition described above, except that no planning questions were asked.

Table S6a. Repeated Measures ANOVA: effects of condition assignment (including an exploratory time-saving voucher condition) and time point on subjective wellbeing.

Source	df	SS	MS	F-value	*p*	η^2
Between-subjects						
Condition	2	0.18	0.09	0.13	0.883	0.000
Error	974	706.00	0.73			
Within-subjects						
Time	2	307.97	153.99	379.99	< 0.001	0.281
Time*Condition	4	2.19	0.55	1.35	0.250	0.003
Error (Time)	1948	789.39	0.41			

Note. Reporting between- and within-subjects effects of condition and time point (baseline, during the intervention, and endline) on subjective wellbeing. Pre-registered conditions only.

Table S6b. Repeated Measures ANOVA: effects of condition assignment (including the exploratory time-saving voucher condition) and time point on subjective wellbeing.

Source	df	SS	MS	F-value	*p*	η^2
Between-subjects						
Condition	2	0.19	0.10	0.13	0.876	0.000
Error	1305	935.64	0.72			
Within-subjects						
Time	2	373.75	186.88	465.48	<0.001	0.263
Time*Condition	4	3.76	0.94	2.34	0.053	0.004
Error (Time)	2610	1047.83	0.41			

Note. Reporting between- and within-subjects effects of condition and time point (baseline, during the intervention, and endline) on subjective wellbeing. Includes exploratory time-saving condition.

Table S6c. Post-hoc contrasts: Time point on subjective wellbeing, collapsing across condition.

Comparison	Mean (SD)	Mean (SD)	Statistics	*d*
Baseline vs. Weeks 3-5 (pre-registered conditions)	2.71 (0.68)	3.48 (0.72)	$t(1026)=27.12, p<0.001$	0.85
Baseline vs. Endline (pre-registered conditions)	2.71 (0.68)	2.89 (0.74)	$t(1070)=6.82, p<0.001$	0.21
Baseline vs. Weeks 3-5 (all time-saving conditions)	2.71 (0.68)	3.49 (0.72)	$t(1376)=31.43, p<0.001$	0.86
Baseline vs. Endline (all time-saving conditions)	2.71 (0.68)	2.88 (0.74)	$t(1437)=7.23, p<0.001$	0.20

Note. "All time-saving conditions" analysis includes the exploratory time-saving condition.

Table S7a. Repeated Measures ANOVA: effects of condition and time on perceived stress

Source	df	SS	MS	F-value	*p*	η^2 [95% CI]
Between-subjects						
Condition	2	1.12	0.56	1.03	0.358	0.003
Error	696	379.10	0.55			
Within-subjects						
Time	2	108.51	54.26	217.42	<0.001	0.238
Time*Condition	4	1.22	0.30	1.22	0.301	0.003
Error (Time)	1392	347.37	0.25			

Note. Reporting between- and within-subjects effects of condition and time point (baseline, during the intervention, and endline) on perceived stress. Pre-registered conditions only.

Table S7b. Repeated Measures ANOVA: effects of condition and time on perceived stress

Source	df	SS	MS	F-value	*p*	η^2 [95% CI]
Between-subjects						
Condition	2	0.75	0.38	0.70	0.499	0.002
Error	924	499.63	0.54			
Within-subjects						
Time	2	135.23	67.62	269.38	<0.001	0.226
Time*Condition	4	2.61	0.65	2.60	0.035	0.006
Error (Time)	1848	463.86	0.25			

Note. Reporting between- and within-subjects effects of condition and time point (baseline, during the intervention, and endline) on perceived stress. Includes exploratory time-saving condition.

Table S7c. Post-hoc contrasts: Time point on subjective wellbeing, collapsing across condition.

Comparison	Mean (SD)	Mean (SD)	Statistics	*d*
Baseline vs. Weeks 3-5 (pre-registered conditions)	3.25 (0.55)	2.68 (0.65)	$t(1003)=23.77, p<0.001$	0.75
Baseline vs. Endline (pre-registered conditions)	3.24 (0.53)	3.06 (0.58)	$t(765)=7.70, p<0.001$	0.29
Baseline vs. Weeks 3-5 (all time-saving conditions)	3.25 (0.55)	2.67 (0.65)	$t(1347)=27.95, p<0.001$	0.77
Baseline vs. Endline (all time-saving conditions)	3.25 (0.54)	3.08 (0.59)	$t(1023)=8.07, p<0.001$	0.24

Note. "All time-saving conditions" analysis includes the exploratory time-saving condition.

Table S8a. Repeated Measures ANOVA: effects of condition and time on relationship conflict

Source	df	SS	MS	F-value	*p*	η^2
Between-subjects						
Condition	2	2.20	1.10	0.94	0.390	0.002
Error	974	1138.78	1.17			
Within-subjects						
Time	2	104.07	52.04	120.99	<0.001	0.110
Time*Condition	4	0.22	0.06	0.13	0.972	0.000
Error (Time)	1948	837.80	0.43			

Note. Reporting between- and within-subjects effects of condition and time point (baseline, during the intervention, and endline) on relationship conflict. Pre-registered conditions only.

Table S8b. Repeated Measures ANOVA: effects of condition and time on relationship conflict

Source	df	SS	MS	F-value	*p*	η^2
Between-subjects						
Condition	2	1.38	0.69	0.59	0.556	0.001
Error	1305	1533.36	1.18			
Within-subjects						
Time	2	122.23	61.11	150.45	<0.001	0.103
Time*Condition	4	0.91	0.23	0.58	0.694	0.001
Error (Time)	2610	1060.23	0.41			

Note. Reporting between- and within-subjects effects of condition and time point (baseline, during the intervention, and endline) on relationship conflict. Includes exploratory time-saving condition.

Table S8c. Post-hoc contrasts: Time point on subjective wellbeing, collapsing across condition.

Comparison	Mean (SD)	Mean (SD)	Statistics	*d*
Baseline vs. Weeks 3-5 (pre-registered conditions)	0.93 (0.95)	0.49 (0.71)	$t(1026)=14.39, p<0.001$	0.45
Baseline vs. Endline (pre-registered conditions)	0.94 (0.96)	0.58 (0.79)	$t(1069)=11.70, p<0.001$	0.36
Baseline vs. Weeks 3-5 (all time-saving conditions)	0.92 (0.93)	0.51 (0.72)	$t(1376)=16.40, p<0.001$	0.44
Baseline vs. Endline (all time-saving conditions)	0.92 (0.95)	0.58 (0.77)	$t(1436)=13.65, p<0.001$	0.35

Note. "All time-saving conditions" analysis includes the exploratory time-saving condition.

Table S9a. Parallel mediation models estimating the indirect effects of the time-saving condition versus the UCT condition on subjective wellbeing, perceived stress, and relationship conflict via total spending (log), cash on hand (log), and perceived burden of unpaid labor.

**Model:**<br>Dependent measure	Mediator	Indirect effect	95% CI
**Model 1:**<br>Subjective wellbeing	Total spending (log)	-0.006<br>(0.004)	-0.001, 0.008
	Cash on hand (log)*	-0.020<br>(0.01)	-0.05, -0.001
	Burden of unpaid labor	0.07<br>(0.06)	-0.04, 0.180
**Model 2:**<br>Perceived stress	Total spending (log)	0.002<br>(0.004)	-0.005, 0.01
	Cash on hand (log)*	0.019<br>(0.01)	0.0008, 0.044
	Burden of unpaid labor*	-0.105<br>(0.05)	-0.19, -0.01
**Model 3:**<br>Relationship conflict	Total spending (log)	-0.003<br>(0.004)	-0.014, 0.003
	Cash on hand (log)	0.004<br>(0.005)	-0.005, 0.016
	Burden of unpaid labor	0.003<br>(0.05)	-0.089, 0.096

Note. Reporting standardized coefficients for 3 parallel mediation models: 1) effects on subjective wellbeing, 2) perceived stress, and relationship conflict. Each of these outcomes are a weighted average of responses during the treatment weeks (weeks 3-5). For each model, the independent variable is condition, where 1=time-saving and 0=UCT. The control condition was dropped from these analyses. The mediators are modelled in parallel, and the following covariates are included in estimates of both the mediator and the dependent measure: baseline income (log) and the respective baseline dependent measure. $N = 620$, listwise deletion of cases with missing data. 5000 bootstrapped samples. *95% confidence interval around the indirect effect does not include 0.

Table S9b. Parallel mediation models estimating the indirect effects of the time-saving condition versus the UCT condition on subjective wellbeing, perceived stress, and relationship conflict via total spending (log), cash on hand (log), and perceived burden of unpaid labor.

**Model:**<br>Dependent measure	Mediator	Indirect effect	95% CI
**Model 1:**<br>Subjective wellbeing	Total spending (log)	-0.0001<br>(0.004)	-0.008, 0.008
	Cash on hand (log)*	-0.020<br>(0.008)	-0.035, -0.004
	Burden of unpaid labor	0.057<br>(0.04)	-0.020, 0.134
**Model 2:**<br>Perceived stress	Total spending (log)	0.0006<br>(0.003)	-0.006, 0.007
	Cash on hand (log)*	0.013<br>(0.007)	0.002, 0.03
	Burden of unpaid labor*	-0.11<br>(0.03)	-0.181, -0.049
**Model 3:**<br>Relationship conflict	Total spending (log)	-0.003<br>(0.004)	-0.01, 0.003
	Cash on hand (log)	0.005<br>(0.005)	-0.004, 0.02
	Burden of unpaid labor	0.03<br>(0.04)	-0.039, 0.099

Note. Reporting standardized coefficients for 3 parallel mediation models: 1) effects on subjective wellbeing, 2) perceived stress, and relationship conflict. Each of these outcomes are a weighted average of responses during the treatment weeks (weeks 3-5). For each model, the independent variable is condition, where 1=time-saving and 0=UCT. Includes exploratory time-saving condition. The control condition was dropped from these analyses. The mediators are modelled in parallel, and the following covariates are included in estimates of the mediator and dependent measure: baseline income (log) and baseline dependent measure. $N = 913$, listwise deletion of cases with missing data. 5000 bootstrapped samples. *95% confidence interval around the indirect effect does not include 0.

Table S10a. Parallel mediation models estimating the indirect effects of the time-saving condition versus the UCT condition on subjective wellbeing, perceived stress, and relationship conflict via percentage time spent on paid work and socializing.

**Model:** Dependent measure	Mediators	Indirect effect	95% CI
**Model 1:** Subjective wellbeing	Time on paid work	0.002 (0.01)	-0.02, 0.02
	Time socializing	0.005 (0.004)	-0.007, 0.013
**Model 2:** Perceived stress	Time on paid work	-0.003 (0.01)	-0.02, 0.02
	Time socializing	-0.009 (0.007)	-0.02, 0.01
**Model 3:** Relationship conflict	Time on paid work	0.0006 (0.004)	-0.006, 0.01
	Time socializing	-0.0007 (0.005)	-0.01, 0.010

Note. Reporting standardized coefficients for 3 parallel mediation models: 1) effects on subjective wellbeing, 2) perceived stress, and relationship conflict. Time spent on paid work and socializing are calculated as a percentage of total time reported in each week; weighted average of the three treatment weeks (weeks 3-5). For each model, the independent variable is condition, where 1=time-saving and 0=UCT. The control condition was dropped from these analyses. The mediators are modelled in parallel, and the respective baseline dependent measure is included as a covariate in estimates of the mediator and dependent measure. N = 520, listwise deletion of cases with missing data. 5000 bootstrapped samples. *95% confidence interval around the indirect effect does not include 0.

Table S10b. Parallel mediation models estimating the indirect effects of the time-saving condition versus the UCT condition on subjective wellbeing, perceived stress, and relationship conflict via percentage time spent on paid work and socializing.

**Model:** Dependent measure	Mediators	Indirect effect	95% CI
**Model 1:** Subjective wellbeing	Time on paid work	0.002 (0.006)	-0.01, 0.01
	Time socializing	-0.002 (0.003)	-0.008, 0.004
**Model 2:** Perceived stress	Time on paid work*	-0.010 (0.004)	-0.002, -0.02
	Time socializing	-0.003 (0.002)	-0.008, 0.0002
**Model 3:** Relationship conflict	Time on paid work	0.002 (0.006)	-0.010, 0.015
	Time socializing	-0.002 (0.003)	-0.008, 0.004

Note. Reporting standardized coefficients for 3 parallel mediation models: 1) effects on subjective wellbeing, 2) perceived stress, and relationship conflict. Time spent on paid work and socializing are calculated as a percentage of total time reported in each week; weighted average of the three treatment weeks (weeks 3-5). For each model, the independent variable is condition, where 1=time-saving and 0=UCT. Includes exploratory time-saving condition. The control condition was dropped from these analyses. The mediators are modelled in parallel, and the respective baseline dependent measure is included as a covariate in estimates of the mediator and dependent measure. *N* = 1033, listwise deletion of cases with missing data. 5000 bootstrapped samples. * 95% confidence interval around the indirect effect does not include 0.

Table S11a. Descriptive statistics and correlations for participants assigned to receive prepared meals ($N = 180$)

Variable	1	2	3	4
1. Subjective wellbeing (1-5) (endline – baseline difference score)	--			
2. Perceived stress (1-5) (endline – baseline difference score)	-.597***	--		
3. Relationship conflict (0-4) (endline – baseline difference score)	-.074	.23*	--	
4. Baseline enjoyment of cooking (1-5)	-.012	0.045	-0.002	--

Notes. Reporting means, standard deviations, and correlations. To measure baseline dislike of cooking, participants responded to the following item: "How much do you enjoy or dislike prepared meals?" Participants provided their response on a 1-5 scale, where 1=very much dislike, 2=somewhat dislike, 3=neither dislike nor enjoy, 4=somewhat enjoy, 5=enjoy very much. Valid N (listwise) = 180. * $p < .05$, ** $p < .01$, ***$p < 0.001$.

Table S11b. Descriptive statistics and correlations for participants assigned to receive prepared meals ($N = 383$)

Variable	1	2	3	4
1. Subjective wellbeing (1-5) (endline – baseline difference score)	--			
2. Perceived stress (1-5) (endline – baseline difference score)	-.522***	--		
3. Relationship conflict (0-4) (endline – baseline difference score)	-0.075	0.085	--	
4. Baseline enjoyment of cooking (1-5)	-0.008	0.054	0.029	--

Notes. Reporting means, standard deviations, and correlations. Includes exploratory time-saving condition. To measure baseline dislike of cooking, participants responded to the following item: "How much do you enjoy or dislike prepared meals?" Participants provided their response on a 1-5 scale, where 1=very much dislike, 2=somewhat dislike, 3=neither dislike nor enjoy, 4=somewhat enjoy, 5=enjoy very much. Valid N (listwise) = 357. * $p < .05$, ** $p < .01$, ***$p < 0.001$.

Table S12a. Descriptive statistics and correlations for participants assigned to receive laundry services ($N = 191$).

Variable	1	2	3	4
1. Subjective wellbeing (1-5) (endline – baseline difference score)	--			
2. Perceived stress (1-5) (endline – baseline difference score)	-.597***	--		
3. Relationship conflict (0-4) (endline – baseline difference score)	-0.074	0.228*	--	
4. Baseline enjoyment of doing laundry (1-5)	0.048	0.065	0.025	--

Notes. Reporting means, standard deviations, and correlations. To measure baseline dislike of laundry, participants responded to the following item: "How much do you enjoy or dislike doing laundry?" Participants provided their response on a 1-5 scale, where 1=very much dislike, 2=somewhat dislike, 3=neither dislike nor enjoy, 4=somewhat enjoy, 5=enjoy very much. Valid N (listwise) = 191. * $p < .05$, ** $p < .01$, *** $p < .001$.

Table S12b. Descriptive statistics and correlations for participants assigned to receive laundry services ($N = 392$).

Variable	1	2	3	4
1. Subjective wellbeing (1-5) (endline – baseline difference score)	--			
2. Perceived stress (1-5) (endline – baseline difference score)	-.540***	--		
3. Relationship conflict (0-4) (endline – baseline difference score)	-.070	0.19*	--	
4. Baseline enjoyment of doing laundry (1-5)	-.118*	.059	0.030	--

Notes. Reporting means, standard deviations, and correlations. Includes exploratory time-saving condition. To measure baseline dislike of laundry, participants responded to the following item: "How much do you enjoy or dislike doing laundry?" Participants provided their response on a 1-5 scale, where 1=very much dislike, 2=somewhat dislike, 3=neither dislike nor enjoy, 4=somewhat enjoy, 5=enjoy very much. Valid N (listwise) = 392. * $p < .05$, ** $p < .01$, *** $p < .001$.

Table S13a. Descriptive statistics and correlations for participants assigned to time-saving conditions (*N* = 378).

Variable	1	2	3	4
1. Subjective wellbeing (1-5) (endline – baseline difference score)	--			
2. Perceived stress (1-5) (endline – baseline difference score)	-.534***	--		
3. Relationship conflict (0-4) (endline – baseline difference score)	-0.073	0.135*	--	
4. Baseline enjoyment of doing chores (1-5)	-0.039	0.045	0.001	--

Notes. Reporting means, standard deviations, and correlations. To measure baseline dislike of doing chores, participants responded to the following item: "How much do you enjoy or dislike completing chores?" Participants provided their response on a 1-5 scale, where 1=very much dislike, 2=somewhat dislike, 3=neither dislike nor enjoy, 4=somewhat enjoy, 5=enjoy very much. Valid *N* (listwise) = 378. * p <0.05, **p <0.01, ***p<0.001.

Table S13b. Descriptive statistics and correlations for participants assigned to time-saving conditions (*N* = 775).

Variable	1	2	3	4
1. Subjective wellbeing (1-5) (endline – baseline difference score)	--			
2. Perceived stress (1-5) (endline – baseline difference score)	-.534***	--		
3. Relationship conflict (0-4) (endline – baseline difference score)	-.073	0.14*	--	
4. Baseline enjoyment of doing chores (1-5)	.025	.049	-0.067	--

Notes. Reporting means, standard deviations, and correlations. Includes exploratory time-saving condition. To measure baseline dislike of completing chores, participants responded to the following item: "How much do you enjoy or dislike completing chores?" Participants provided their response on a 1-5 scale, where 1=very much dislike, 2=somewhat dislike, 3=neither dislike nor enjoy, 4=somewhat enjoy, 5=enjoy very much. Valid *N* (listwise) = 358. *p <0.05, **p <0.01, ***p<0.001.

Table S14a. Moderation analyses predicting endline effects of the time-saving vs. UCT condition

Dependent measure	Moderator	Interaction statistics
Subjective wellbeing	Education	$R^2\Delta$=.000, F(1, 699)=0.080, p=0.777
	Occupation	$R^2\Delta$=.005, F(4, 692)=0.923, p=0.450
	Micro-enterprise ownership	$R^2\Delta$=.003, F(1, 710)=1.97, p=0.161
	Household size	$R^2\Delta$=.001, F(1, 710)=0.929, p=0.336
	Baseline monthly income (log)	$R^2\Delta$=.0001, F(1, 706)=0.055, p=0.815
	Baseline subjective wellbeing	$R^2\Delta$=.000, F(1, 711)=0.034, p=0.854
	Baseline perceived stress	$R^2\Delta$=.003, F(1, 711)=2.38, p=0.123
	Baseline relationship conflict	$R^2\Delta$=.000, F(1, 711)=0.006, p=0.940
	Baseline CES-D	$R^2\Delta$=.000, F(1, 711)=0.012, p=0.910
Perceived stress	Education	$R^2\Delta$=.000, F(1, 698)=0.175, p=0.676
	Occupation	$R^2\Delta$=.004, F(4, 691)=0.687, p=0.601
	Micro-enterprise ownership***	$R^2\Delta$=.015, F(1, 709)=11.25, p<.001
	Household size	$R^2\Delta$=.002, F(1, 709)=1.25, p=0.264
	Baseline monthly income (log)	$R^2\Delta$=.0001, F(1, 705)=0.094, p=0.759
	Baseline subjective wellbeing	$R^2\Delta$=.000, F(1, 709)=0.032, p=0.858
	Baseline perceived stress	$R^2\Delta$=.000, F(1, 710)=0.064, p=0.801
	Baseline relationship conflict	$R^2\Delta$=.000, F(1, 710)=0.003, p=0.959
	Baseline CES-D	$R^2\Delta$=.0003, F(1, 710)=0.226, p=0.635
Relationship conflict	Education	$R^2\Delta$=.0006, F(1, 699)=0.499, p=0.480
	Occupation	$R^2\Delta$=.005, F(4, 692)=1.14, p=0.335
	Micro-enterprise ownership	$R^2\Delta$=.003, F(1, 710)=2.37, p=0.124
	Household size	$R^2\Delta$=.0003, F(1, 710)=0.245, p=0.621
	Baseline monthly income (log)	$R^2\Delta$=.000, F(1, 706)=0.019, p=0.892
	Baseline subjective wellbeing	$R^2\Delta$=.000, F(1, 710)=0.530, p=0.467
	Baseline perceived stress	$R^2\Delta$=.002, F(1, 710)=2.08, p=0.149
	Baseline relationship conflict	$R^2\Delta$=.001, F(1, 711)=1.18, p=0.278
	Baseline CES-D	$R^2\Delta$=.0004, F(1, 711)=0.285, p=0.593

Note. Reporting 27 moderation analyses, each using Preacher and Hayes Process model 1 with 5000 bootstrapped samples. All models control for the respective baseline dependent measure. Occupation was coded into 4 dummy variables: 1) trades, 2) personal services, 3) casual labor, and 4) childcare, education, and healthcare services; 'all sales jobs' coded as the reference category. To adjust for multiple comparisons, we used a Bonferroni correction. With this correction, the significance level for each comparison is $p \leq 0.002$. Thus, micro-enterprise ownership was the only individual difference that influenced that effect of time-saving vouchers versus UCTs. ***p < .001.

Table S14b. Moderation analyses predicting endline effects of the time-saving vs. UCT condition

Dependent measure	Moderator	Interaction statistics
Subjective wellbeing	Education	$R^2\Delta$=.000, F(1, 1061)=0.399, p=0.528
	Occupation	$R^2\Delta$=.004, F(4, 1049)=1.21, p=0.303
	Micro-enterprise ownership	$R^2\Delta$=.004, F(1, 1077)=4.29, p=0.039
	Household size	$R^2\Delta$=.002, F(1, 1077)=2.02, p=0.156
	Baseline monthly income (log)	$R^2\Delta$=.000, F(1, 1070)=0.005, p=0.943
	Baseline subjective wellbeing	$R^2\Delta$=.000, F(1, 1078)=0.498, p=0.481
	Baseline perceived stress	$R^2\Delta$=.005, F(1, 1077)=6.31, p=0.012
	Baseline relationship conflict	$R^2\Delta$=.000, F(1, 1077)=0.486, p=0.486
	Baseline CES-D	$R^2\Delta$=.000, F(1, 1077)=0.338, p=0.561
Perceived stress	Education	$R^2\Delta$=.000, F(1, 1059)=0.085, p=0.771
	Occupation	$R^2\Delta$=.003, F(4, 1047)=0.894, p=0.467
	Micro-enterprise ownership***	$R^2\Delta$=.014, F(1, 1075)=16.24, p=0.0001
	Household size	$R^2\Delta$=.000, F(1, 1075)=0.490, p=0.484
	Baseline monthly income (log)	$R^2\Delta$=.000, F(1, 1068)=0.424, p=0.515
	Baseline subjective wellbeing	$R^2\Delta$=.000, F(1, 1075)=0.102, p=0.750
	Baseline perceived stress	$R^2\Delta$=.000, F(1, 1076)=0.032, p=0.859
	Baseline relationship conflict	$R^2\Delta$=.000, F(1, 1075)=0.421, p=0.517
	Baseline CES-D	$R^2\Delta$=.000, F(1, 1075)=0.360, p=0.548
Relationship conflict	Education	$R^2\Delta$=.000, F(1, 1061)=1.124, p=0.289
	Occupation	$R^2\Delta$=.005, F(4, 1049)=1.503, p=0.199
	Micro-enterprise ownership	$R^2\Delta$=.002, F(1, 1077)=2.052, p=0.152
	Household size	$R^2\Delta$=.000, F(1, 1077)=0.107, p=0.744
	Baseline monthly income (log)	$R^2\Delta$=.000, F(1, 1070)=0.597, p=0.440
	Baseline subjective wellbeing	$R^2\Delta$=.000, F(1, 1077)=0.078, p=0.780
	Baseline perceived stress	$R^2\Delta$=.000, F(1, 1077)=0.644, p=0.423
	Baseline relationship conflict	$R^2\Delta$=.000, F(1, 1078)=0.985, p=0.321
	Baseline CES-D	$R^2\Delta$=.0004, F(1, 1078)=0.519, p=0.519

Note. Reporting 27 moderation analyses, each using Preacher and Hayes Process model 1 with 5000 bootstrapped samples. Includes exploratory time-saving condition. All models control for the respective baseline dependent measure. Occupation was coded into 4 dummy variables with ‘all sales jobs’ coded as the reference category. To adjust for multiple comparisons, we used a Bonferroni correction. With this correction, the significance level for each comparison is $p \leq 0.002$. Thus, micro-enterprise ownership was the only individual difference that influenced that effect of time-saving vouchers versus UCTs. *** $p < .001$.

Follow-up on significant interaction effects predicting endline perceived stress

Table S15a. Moderation analysis conditional effects: effect of time-saving vs UCT on endline perceived stress, controlling for baseline, conditional on micro-enterprise ownership

Conditional effects	*β*	*t*	*p*	95% CI (*β*) Lower bound	Upper bound
Micro-enterprise ownership=0	-0.08 (0.06)	-1.49	0.138	-0.192	0.027
Micro-enterprise ownership=1	0.23 (0.07)	3.09	0.002	0.082	0.369

Note. Reporting standardized coefficients, *t*-statistics, *p*-values, and 95% confidence intervals. 5000 bootstrapped samples. $N = 714$.

Table S15b. Moderation analysis conditional effects: effect of time-saving vs UCT on endline perceived stress, controlling for baseline, conditional on micro-enterprise ownership

Conditional effects	*β*	*t*	*p*	95% CI (*β*) Lower bound	Upper bound
Micro-enterprise ownership=0	-0.08 (0.05)	-1.57	0.117	-0.169	0.019
Micro-enterprise ownership=1	0.24 (0.06)	3.866	<0.001	0.121	0.369

Note. Reporting standardized coefficients, *t*-statistics, *p*-values, and 95% confidence intervals. 5000 bootstrapped samples. $N = 1080$. Includes exploratory time-saving condition.

Table S16. Exclusions based on pre-registered criteria from initial participant pool

**Variable**	**Decision Rule**	**# of Exclusions**	**Remaining Eligible**
Lives in Kibera	If no, exclude.	360 of 4,286 (8.4%)	3,926[a]
Available to participate	If no, exclude.	475 of 3,926 (12.1%)	3,622[a]
Consent Completed	If no, exclude.	22 of 3,622 (0.06%)	3,600
Gender	If male, exclude.	6 of 3,600 (0.02%)	3,600[b]
Children	If no, exclude.	84 of 3,600 (1.9%)	3,510
Children Living at Home	If no, exclude.	103 of 3,510 (2.3%)	3,407
Children Enrolled in School	If no, exclude.	142 of 3,407 (3.2%)	3,265
25+ hours worked / week	If no, exclude.	549 of 3,265 (16.8%)	2,716
Completed their own laundry	If no, exclude.	9 of 2,716 (0.3%)	2,707
Spent fewer than 3 hours on laundry each week	If yes, exclude.	1 of 2,707 (0.0%)	2,706
"Always" paid money for someone else to do laundry	If yes, exclude.	4 of 2,706 (0.01%)	2,702
Completed own cooking	If no, exclude.	0 of 2,702 (0.0%)	2,702
Spent fewer than 3 hours cooking each week	If yes, exclude.	0 of 2,702 (0.0%)	2,702
"Always" paid money for someone to cook for them	If yes, exclude.	1 of 2,702 (0.0%)	2,701
7 or more people living in the participants' household?	If yes, exclude.	71 of 2,701 (2.6%)	2,630
Travel to Kibera Town Center was 45 minutes or more	If yes, exclude.	21 of 2,630 (0.8%)	2,609[c]
Participant or household member had food allergies	If yes, exclude.	36 of 2,609 (2.4%)	2,573[d]
TOTAL ELIGIBLE:			2,573 (57.7%)

Note: To save time and money, the eligibility survey was designed such that if a participant was excluded on any variable, the survey would be terminated immediately. For example, if a participant did not live in Kibera, they were not asked if they were free to participate. Thus, the number of exclusions reflect participants who were excluded on each variable (after inclusion on the previous variables). [a]A subset of participants (N =171) did not answer the first two questions and were treated as missing in these analyses. [b]The survey did not automatically exclude participants after they reported their gender, explaining the identical denominator for children and gender in this table. [c]We decided to exclude participants only if they lived 45 minutes or farther from KTC (vs. 30 minutes as per our pre-registration) based on recruitment advice from our field officers. Although many participants lived more than 30 minutes away, they passed by Kibera Town Center frequently while commuting to work or running errands, thus KTC was conveniently located for most. [d]A subset of participants did not answer this question (N =358).

Table S17. Characteristics of participants who completed the endline survey in-person (n = 764) versus over the phone (n = 306).

Variable (measured at baseline)	In-person endline Mean (SD)	Phone endline Mean (SD)	Difference statistics
Age	34.09 (8.16)	36.90 (9.64)	$t(1065) = -4.56, p < .001$
Education (% completed primary school)	77%	82%	$X^2(N=1, 1070) = 2.99, p = .10$
% married or marriage-like relationship	60%	59%	$X^2(N=1, 1070) = 0.089, p = .78$
Household size (total # of people)	4.73 (1.57)	4.56 (1.49)	$t(1068) = -1.59, p = .12$
Number of children in the household	3.09 (1.44)	2.92 (1.421)	$t(1068) = -1.82, p = .07$
% responsible for financial decisions	50%	49%	$X^2(N=1, 1070) = 0.09, p = .79$
Hours of paid labor in past 7 days	43.40 (20.49)	46.99 (21.36)	$t(1068) = 2.56, p = .01$
Hours of unpaid labor in past 7 days	39.21 (24.26)	44.72 (25.23)	$t(1068) = 3.31, p = .001$
Personal income in past 6 months	KSH 38,696 (46,417)	KSH 43,025 (49,308)	$t(1059) = 1.75, p = .08$
Household spending in the past 7 days	KSH 3,199 (3,563)	KSH 3,891 (4,198)	$t(1068) = 2.72, p = .01$
Baseline depression (CES-D; 1- 4)	2.24 (0.47)	2.24 (0.51)	$t(1068) = -0.07, p = .94$
Baseline SWB (1 – 5)	2.73 (0.68)	2.65 (0.68)	$t(1068) = -1.58, p = .12$
Baseline PSS (1 – 5)	3.24 (0.53)	3.24 (0.56)	$t(1068) = 0.11, p = .91$
Baseline relationship conflict[a] (0 – 4)	0.95 (0.96)	0.91 (0.97)	$t(1068) = -0.61, p = .54$

Notes. Reporting means and standard deviations for respondent characteristics at baseline. To adjust for multiple comparisons, we have used Bonferroni correction. Using this correction, the significance level for these comparisons is $p < 0.004$. Therefore, participants who completed the endline survey over the phone differ only in terms of age and baseline hours of unpaid labor as compared to those who completed the endline survey in-person at KTC.

Table S18. Bayesian model comparison on the subset of participants who completed the endline survey in-person at KTC (excluding those who completed the over the phone and therefore could not complete the Satisfaction with Life measure).

Models	P(M)	P(M|data)	BF_M	BF_{10}	error %
**Models predicting endline SWB:**					
M_0: Null model (incl. baseline SWB)	0.500	0.896	8.644	1.000	
M_1: Condition + baseline SWB	0.500	0.104	0.116	0.116	5.247

Note: Reporting the prior model probability, P(M); the posterior model probability, P(M|data); the posterior model odds, BF_M; and the Bayes Factor indicating the predictive performance of a given model divided by the predictive performance of the null model (BF_{10}). $N = 764$.

Table S19. Bayesian pairwise comparisons on the subset of participants who completed the endline survey in-person at KTC.

		Prior Odds	Posterior Odds	BF_{10}	error %
**Pairwise comparisons on endline SWB**					
Control	UCT	0.587	0.058	0.098	< .001
Control	Time-saving	0.587	0.174	0.297	< .001
UCT	Time-saving	0.587	0.213	0.363	< .001

Note: Individual comparisons are based on a Cauchy prior distribution with an *r*-scale value of 0.3 for comparisons between the UCT condition and the control condition; 0.5 for comparisons between the Time-saving condition and the control condition; and 0.4 for comparisons between the UCT and Times-saving conditions. $N = 764$.

Table S20. Bayesian model comparison on the subset of participants who reported being married or in a marriage-like relationship (and therefore responded to the relationship conflict questions with respect to their romantic partner)

Models	P(M)	P(M|data)	BF_M	BF_{10}	error %
**Models predicting endline conflict:**					
M_0: Null model (incl. baseline conflict)	0.500	0.927	12.635	1.000	
M_1: Condition + baseline conflict	0.500	0.073	0.079	0.079	2.705

Note: Reporting the prior model probability, P(M); the posterior model probability, P(M|data); the posterior model odds, BF_M; and the Bayes Factor indicating the predictive performance of a given model divided by the predictive performance of the null model (BF_{10}). $N = 604$.

Table S21. Bayesian pairwise comparisons on the subset of participants who reported being married or in a marriage-like relationship

		Prior Odds	Posterior Odds	BF_{10}	error %
**Pairwise comparisons on endline conflict**					
Control	UCT	0.587	0.247	0.421	< .001
Control	Time-saving	0.587	0.327	0.557	.007
UCT	Time-saving	0.587	0.066	0.112	< .001

Note: Individual comparisons are based on a Cauchy prior distribution with an *r*-scale value of 0.3 for comparisons between the UCT condition and the control condition; 0.5 for comparisons between the Time-saving condition and the control condition; and 0.4 for comparisons between the UCT and Times-saving conditions. $N = 604$.
